# Critical analysis of the prescription and evaluation of protein kinase inhibitors for oncology in Germany

**DOI:** 10.1007/s00210-023-02475-9

**Published:** 2023-04-04

**Authors:** Caecilia S. Obst, Roland Seifert

**Affiliations:** Institute of Pharmacology, Hannover Medical School, Carl-Neuberg-Str. 1, 30625 Hannover, Germany

**Keywords:** Oncology, Targeted therapeutics, Early benefit assessment, Pharmaceutical costs, Health economics, AVR

## Abstract

The prices of oncology drugs have been rising progressively in recent years. Despite accounting for only a small share of prescriptions, oncology drugs are the most expensive drugs on the market. However, the association between drug price and clinical benefit often remains questionable. Therefore, we set out to analyze the development of prescription and benefit assessment of protein kinase inhibitors. We identified 20 protein kinase inhibitors with oncological indications that were newly approved by the *European Medicines Agency* (EMA) between 2015 and 2019, based on the *Arzneiverordnungsreport* (AVR, *Drug Prescription Report*). For these 20 drugs, the number of prescriptions, sales, defined daily dose (DDD), and DDD costs were identified for the year of approval and for 2020, respectively, based on data from the *Wissenschaftliches Institut der Ortskrankenkassen* (WIdO, *Scientific Institute of the General Local Health Insurance Fund, AOK*). Moreover, the additional benefit assessments by the *Gemeinsamer Bundesausschuss* (GBA, *Federal Joint Committee*) were considered for each drug. It is shown that the share of a drug in prescriptions, sales, and DDD does not correlate with the clinical benefit of the drug as measured by the additional benefit assessment by the GBA. Lastly, the advertisement pattern of protein kinase inhibitors in a representative oncology journal does not correlate with drug benefit. In conclusion, the immense costs of oncology drugs are therefore largely caused by drugs for which no additional benefit has been proven by the GBA. In order to ensure the long-term stability of health care systems, price-regulation measures are urgently needed, especially for drugs whose additional benefit has not been proven.

**Supplementary Information:**

The online version contains supplementary material available at 10.1007/s00210-023-02475-9.

## Introduction

The pharmaceutical expenditures of the *Gesetzliche Krankenversicherung* (GKV, statutory health insurance, SHI) are increasing progressively. In 2020, GKV drug expenditures amounted to 45.570 billion euros, which corresponds to an increase of 5.1% compared to the previous year. Despite only accounting for a small share of prescriptions (1.2%), the oncology drug group accounted for the highest costs in the GKV drug market with 9.457 billion euros. Within the group of oncology drugs, the high costs are primarily due to the very expensive “targeted therapeutics,” i.e., monoclonal antibodies (€3.715 billion) and protein kinase inhibitors (€2.391 billion) (Ludwig et al. 2021). However, the association between drug price and clinical benefit of a drug remains questionable. In our analysis, we focused on the drug group of protein kinase inhibitors. As a type of targeted therapeutics, protein kinase inhibitors target specific changes in tumor cells for antineoplastic effects. Therefore, they cause less damage to normal cells than the classic cytostatic drugs. Since the importance of this targeted tumor therapy in oncology is increasing, we focused on the protein kinase inhibitors in this analysis and aimed to analyze the development of prescriptions and benefit assessments of these inhibitors.

## Materials and methods

In the following analysis, based on the 2016 to 2020 *Arzneiverordnungsreport* (AVR, *Drug Prescription Report*), the new protein kinase inhibitors for oncological indications, approved by the *European Medicines Agency* (EMA) between 2015 and 2019, were identified (Schwabe and Paffrath 2016; Paffrath et al. 2017; Schwabe et al. 2018; Schwabe et al. 2019; Schwabe and Ludwig 2020). Only new drugs were considered whereas already known protein kinase inhibitors with new indications or in new combinations were not. In addition, the drugs were classified according to the official anatomical-therapeutic-chemical classification with daily doses for the German drug market 2021 (ATC-Index 2021). According to this, only drugs that are classified under L01E as protein kinase inhibitors were examined (Bundesinstitut für Arzneimittel und Medizinprodukte (BfArM), 2021). Taking these criteria into account, 20 drugs could be identified. Three of these drugs (lenvatinib, midostaurin, and gilteritinib) were approved as orphan drugs, i.e. drugs that are used for the treatment of orphan diseases. An overview of the analyzed drugs is shown in Table 1.

**Table 1:** Overview of analyzed drugs and indications

	Drug (trade name)	Launch, indication
1a	Ceritinib (Zykadia^®^)	**2015:** ALK-positive, advanced NSCLC, previously treated with crizotinib
1b	Ceritinib (Zykadia^®^)	**2017:** ALK-positive, advanced NSCLC, first-line treatment
2	Cobimetinib (Cotellic^®^)	**2015:** Metastatic melanoma with BRAF V600 mutation
3a	Lenvatinib (Lenvima^®^)	**2015:** Metastatic thyroid carcinoma
3b	Lenvatinib (Lenvima^®^)	**2016:** Advanced renal cell carcinoma
3c	Lenvatinib (Lenvima^®^)	**2018:** Advanced or unresectable hepatocellular carcinoma
3d	Lenvatinib (Lenvima^®^)	**2021:** Endometrial carcinoma, previously treated with platinum-containing therapy, in combination with pembrolizumab
3e	Lenvatinib (Lenvima^®^)	**2021:** Advanced renal cell carcinoma, first-line treatment, in combination with pembrolizumab
4	Nintedanib (Vargatef^®^)	**2015:** Metastatic NSCLC
5a	Trametinib (Mekinist^®^)	**2015:** Melanoma with BRAF V600 mutation, in combination with dabrafenib
5b	Trametinib (Mekinist^®^)	**2017:** Advanced NSCLC with BRAF V600 mutation, in combination with dabrafenib
6a	Osimertinib (Tagrisso^®^)	**2016:** Metastatic NSCLC with T790M-EGFR mutation
6b	Osimertinib (Tagrisso^®^)	**2019:** Metastatic NSCLC with T790M-EGFR mutation, first-line treatment
6c	Osimertinib (Tagrisso^®^)	**2021:** Metastatic NSCLC with T790M-EGFR mutation, adjuvant treatment
7	Palbociclib (Ibrance^®^)	**2016:** Hormone receptor-positive, HER2-negative, locally advanced or metastatic breast cancer
8a	Alectinib (Alecensa^®^)	**2017:** ALK-positive, advanced NSCLC, previously treated with crizotinib
8b	Alectinib (Alecensa^®^)	**2017:** ALK-positive, advanced NSCLC, first-line treatment
9a	Midostaurin (Rydapt^®^)	**2017:** AML with FLT3 mutation
9b	Midostaurin (Rydapt^®^)	**2017:** Aggressive systemic mastocytosis, systemic mastocytosis with associated hematological neoplasm or mast cell leukemia
10a	Ribociclib (Kisqali^®^)	**2017:** Hormone receptor-positive, HER2-negative, locally advanced or metastatic breast cancer, in combination with an aromatase inhibitor
10b	Ribociclib (Kisqali^®^)	**2017:** Hormone receptor-positive, HER2-negative, locally advanced or metastatic breast cancer, in combination with fulvestrant
11	Tivozanib (Fotivda^®^)	**2017:** Advanced renal cell carcinoma, first-line treatment
12a	Abemaciclib (Verzenios^®^)	**2018:** Hormone receptor-positive, HER2-negative, locally advanced or metastatic breast cancer, in combination with an aromatase inhibitor
12b	Abemaciclib (Verzenios^®^)	**2018:** Hormone receptor-positive, HER2-negative, locally advanced or metastatic breast cancer, in combination with fulvestrant
13	Binimetinib (Mektovi^®^)	**2018:** Melanoma with BRAF V600 mutation, in combination with encorafenib
14a	Encorafenib (Braftovi^®^)	**2018:** Melanoma with BRAF V600 mutation, in combination with binimetinib
14b	Encorafenib (Braftovi^®^)	**2020:** Metastatic colorectal cancer with BRAF V600 mutation after prior systemic therapy, in combination with cetuximab
15a	Brigatinib (Alunbrig^®^)	**2019:** ALK-positive, advanced NSCLC, previously treated with crizotinib
15b	Brigatinib (Alunbrig^®^)	**2020:** ALK-positive, advanced NSCLC, previously not treated with an ALK inhibitor, with brain metastases
15c	Brigatinib (Alunbrig^®^)	**2020:** ALK-positive, advanced NSCLC, previously not treated with an ALK inhibitor, without brain metastases
16	Dacomitinib (Vizimpro^®^)	**2019:** NSCLC with EGFR-activating mutations, first-line treatment
17	Gilteritinib (Xospata^®^)	**2019:** AML with FLT3 mutation
18	Larotrectinib (Vitrakvi^®^)	**2019:** Tumors that display a Neurotrophic Tyrosine Receptor Kinase gene fusion
19	Lorlatinib (Lorviqua^®^)	**2019:** ALK-positive, advanced NSCLC
20	Neratinib (Nerlynx^®^)	**2019:** Hormone receptor-positive, HER2-overexpressed/amplified breast cancer

### Prescription data

For the 20 drugs, the number of prescriptions, sales, the defined daily dose (DDD) and the DDD costs were determined on the basis of data from the *Wissenschaftliches Institut der Ortskrankenkassen* (WIdO, *Scientific Institute of the General Local Health Insurance Fund, AOK*) (https://www.wido.de/, last accessed November 5, 2022). In each case, the values from the year of approval of the drug were compared with the values in 2020. The data refer to drugs prescribed by physicians for outpatient use and dispensed via public pharmacies at the expense of the GKV system. Due to changes in classification or DDD, there may be deviations from the AVR data. Table S1 provides an overview of the prescription data determined.

### Additional benefit assessment

Furthermore, the additional benefit assessment was determined by the *Gemeinsamer Bundesausschuss* (GBA, *Federal Joint Committee)* for each drug (https://www.g-ba.de/, last accessed November 5, 2022). Compared to the appropriate comparative (standard) therapy, the GBA differentiated between six categories for the additional benefit assessment of a drug: major additional benefit, considerable additional benefit, minor additional benefit, not quantifiable additional benefit, no additional benefit, and less benefit (Gemeinsamer Bundesausschuss 2022). The initial additional benefit assessment of each drug for the indication of the marketing authorization was compared with all further additional benefit assessments for the respective drug published by the GBA until February 2022. Thus, re-evaluations after the deadline of the initial evaluation, as well as additional benefit evaluations for new indications for the individual 20 drugs that resulted after the approval, were determined. As a result, a total of 41 additional benefit assessments by the GBA could be identified for the 20 drugs in 33 indications. Based on the benefit assessments of the *Institut für Qualität und Wirtschaftlichkeit im Gesundheitswesen* (IQWiG, *Institute for Quality and Efficiency in Health Care*) on which the GBA based its additional benefit assessment, all relevant studies on which the benefit assessment was based were identified (https://www.iqwig.de/, last accessed November 5, 2022). In a further step, additional relevant studies published until March 28, 2022, were identified for the respective drugs through literature searches in the PubMed database (https://pubmed.ncbi.nlm.nih.gov/, last accessed November 5, 2022). An overview of the analyzed additional benefit assessments is shown in Table S2. Table S3 shows the pharmacological characterization of the analyzed drugs.

Finally, the additional benefit assessments by the GBA for the four drugs with the largest share of prescriptions, sales, and DDD in this analysis were compared with the drug assessments by the *European Society for Medical Oncology* (ESMO) and the *Deutsche Gesellschaft für Hämatologie und Onkologie* (DGHO, *German Society for Hematology and Oncology*) and similarities and differences were analyzed (https://www.esmo.org/guidelines/esmo-mcbs/esmo-mcbs-scorecards and https://www.dgho.de/publikationen/stellungnahmen/fruehe-nutzenbewertung, last accessed November 7, 2022).

### Advertisements

In addition, the advertisements published in the oncological journal *Oncology Research and Treatment* were analyzed exemplarily (https://www.karger.com/Journal/Home/224106, last accessed November 5, 2022). For this purpose, all 10 issues of the journal from 2020 were examined with regard to published advertisements on drugs.

## Results

### Prescriptions

**Fig. 1:**
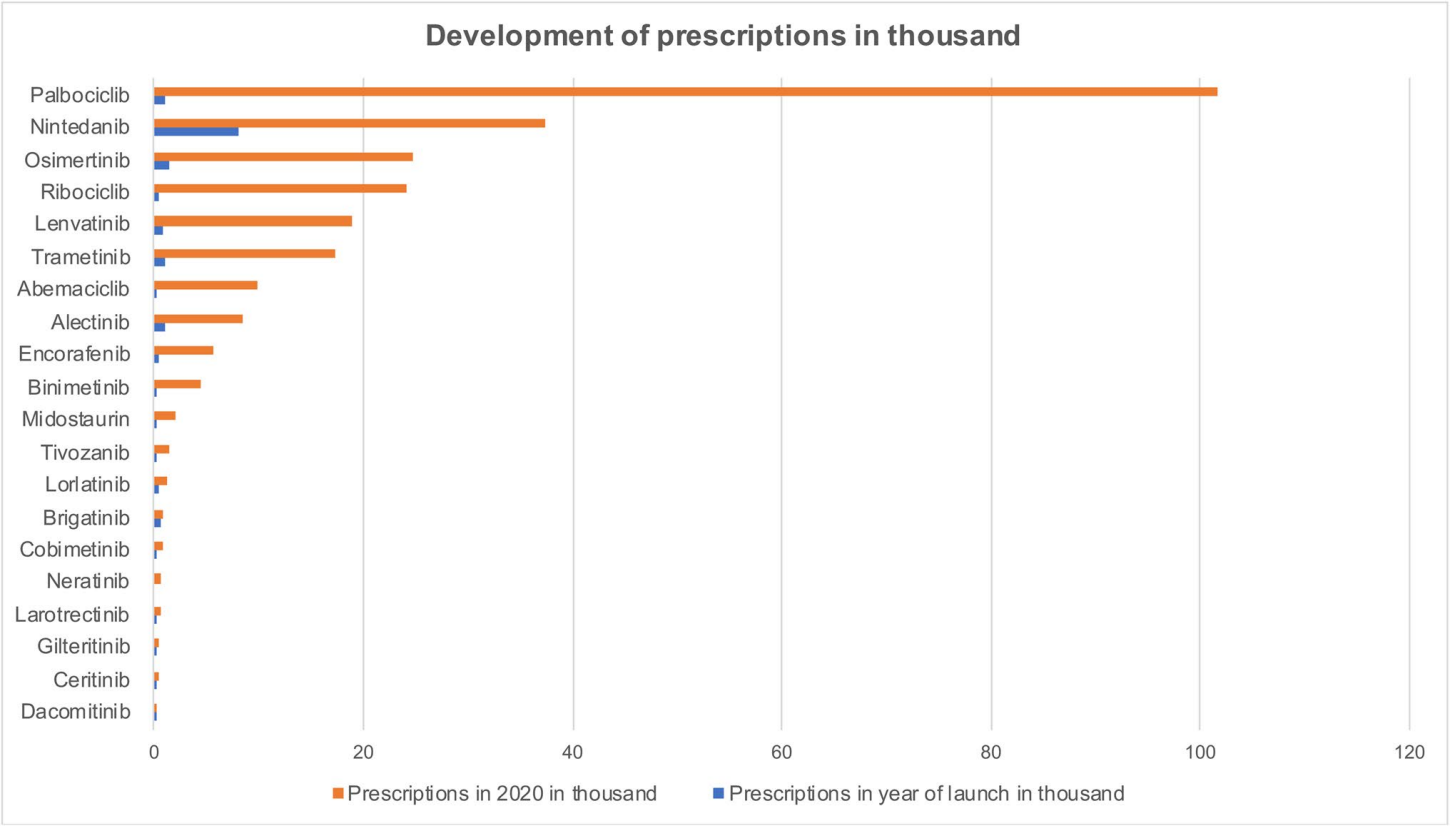
Development of prescriptions in thousand

Comparing the year of introduction and 2020, the number of prescriptions increased for each of the 20 drugs (Fig. 1). Palbociclib had the largest number of prescriptions in 2020 with 101.64 thousand prescriptions, followed by nintedanib with 37.37 thousand prescriptions, osimertinib with 24.65 thousand prescriptions, and ribociclib with 24.11 thousand prescriptions.

### Sales

**Fig. 2:**
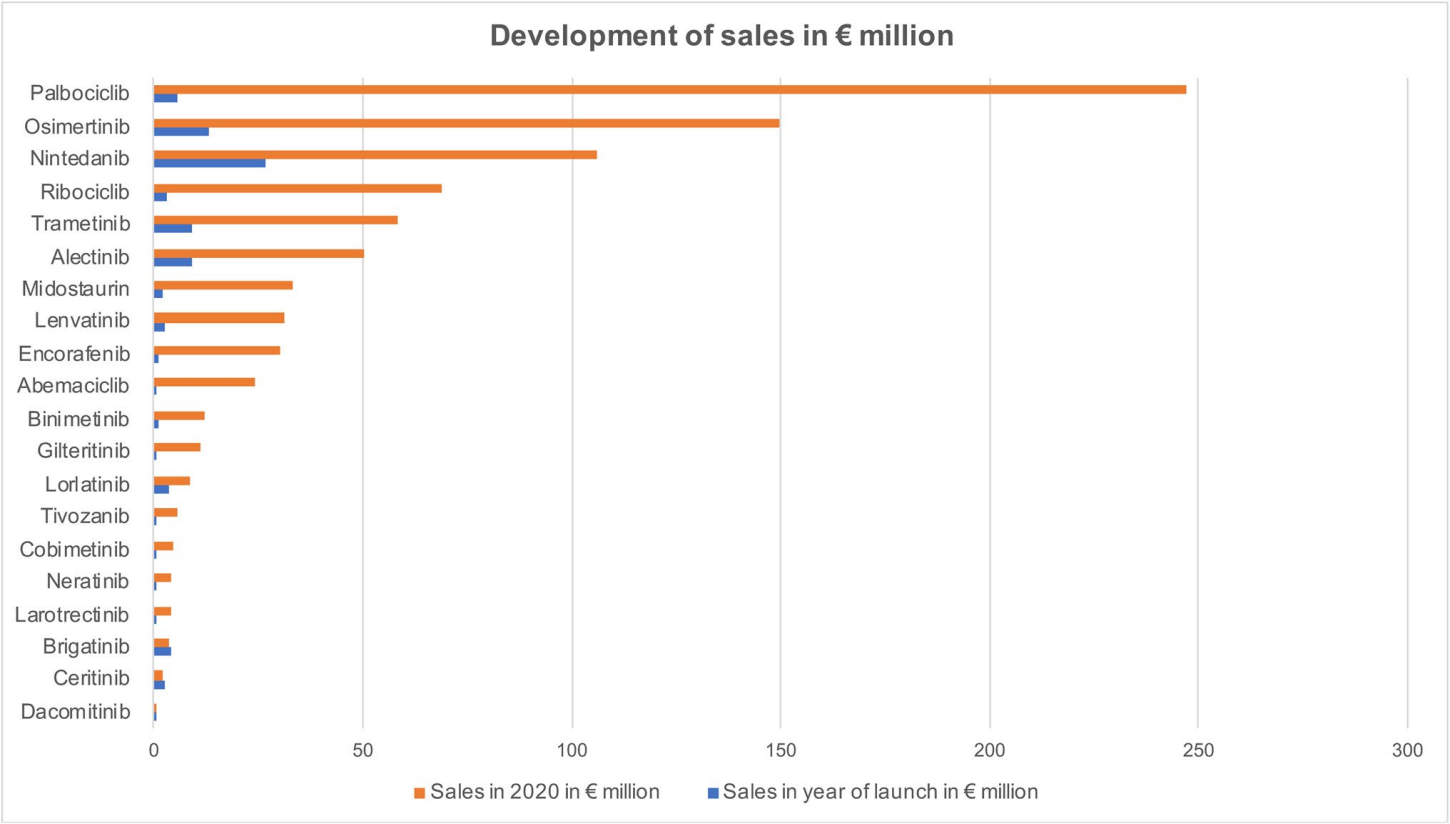
Development of sales in € million

Sales increased for 18 of the 20 drugs when comparing the year of introduction and 2020 (Fig. 2). It decreased for the two anaplastic lymphoma kinase (ALK) inhibitors brigatinib and ceritinib. The highest sales in 2020 were also achieved by the four drugs palbociclib (€247.09 million), osimertinib (€149.72 million), nintedanib (€106.00 million), and ribociclib (€68.80 million).

### DDD

DDDs increased for 19 of the 20 drugs when comparing the year of introduction and 2020. They decreased for ceritinib only. However, palbociclib (2457.70 thousand), nintedanib (990.50 thousand), ribociclib (743.50 thousand), and osimertinib (676.00 thousand) had the most DDDs.

### DDD costs

DDD costs are calculated by dividing net costs by DDD. For all drugs except ceritinib, both net costs and DDD increased, thus DDD costs decreased. For ceritinib, on the other hand, net costs and DDD decreased, and DDD costs increased. 

### General development

The four drugs palbociclib, nintedanib, osimertinib, and ribociclib accounted for the largest share of prescriptions, sales, and DDD in this analysis. In the following, these will be referred to the *Top 4.* Palbociclib and ribociclib are CDK inhibitors used for treatment of hormone receptor positive, HER2-negative locally advanced or metastatic breast cancer. The angiokinase inhibitor nintedanib is used for treatment of metastatic NSCLC and EGFR inhibitor osimertinib is used for treatment of metastatic NSCLC with T790M-EGFR-mutation. The five drugs ceritinib, brigatinib, dacomitinib, gilteritinib, and larotrectinib had the lowest share of prescriptions, sales, and DDD in this analysis and are therefore referred to the *Flop 5* below*.*

### Initial GBA benefit assessment

**Fig. 3:**
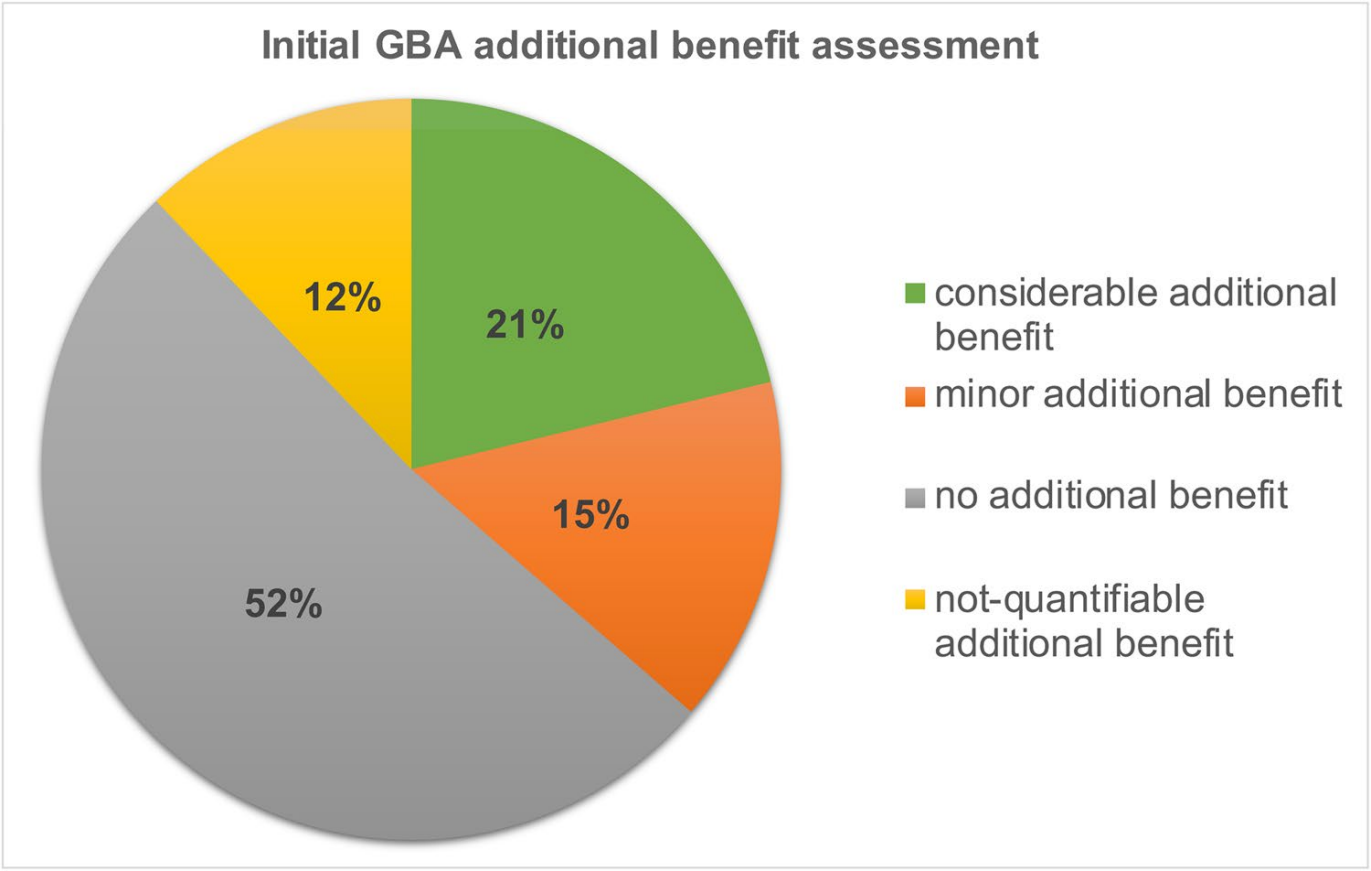
Initial GBA additional benefit assessment

In the initial additional benefit assessment by the GBA, no additional benefit could be determined for 52% of the 20 drugs in 33 indications. Fifteen percent of the 20 drugs were assessed with a minor additional benefit, 21% had a considerable additional benefit, and for 12%, the additional benefit could not be quantified (Fig. 3).

### 2020 current (re)assessment

**Fig. 4:**
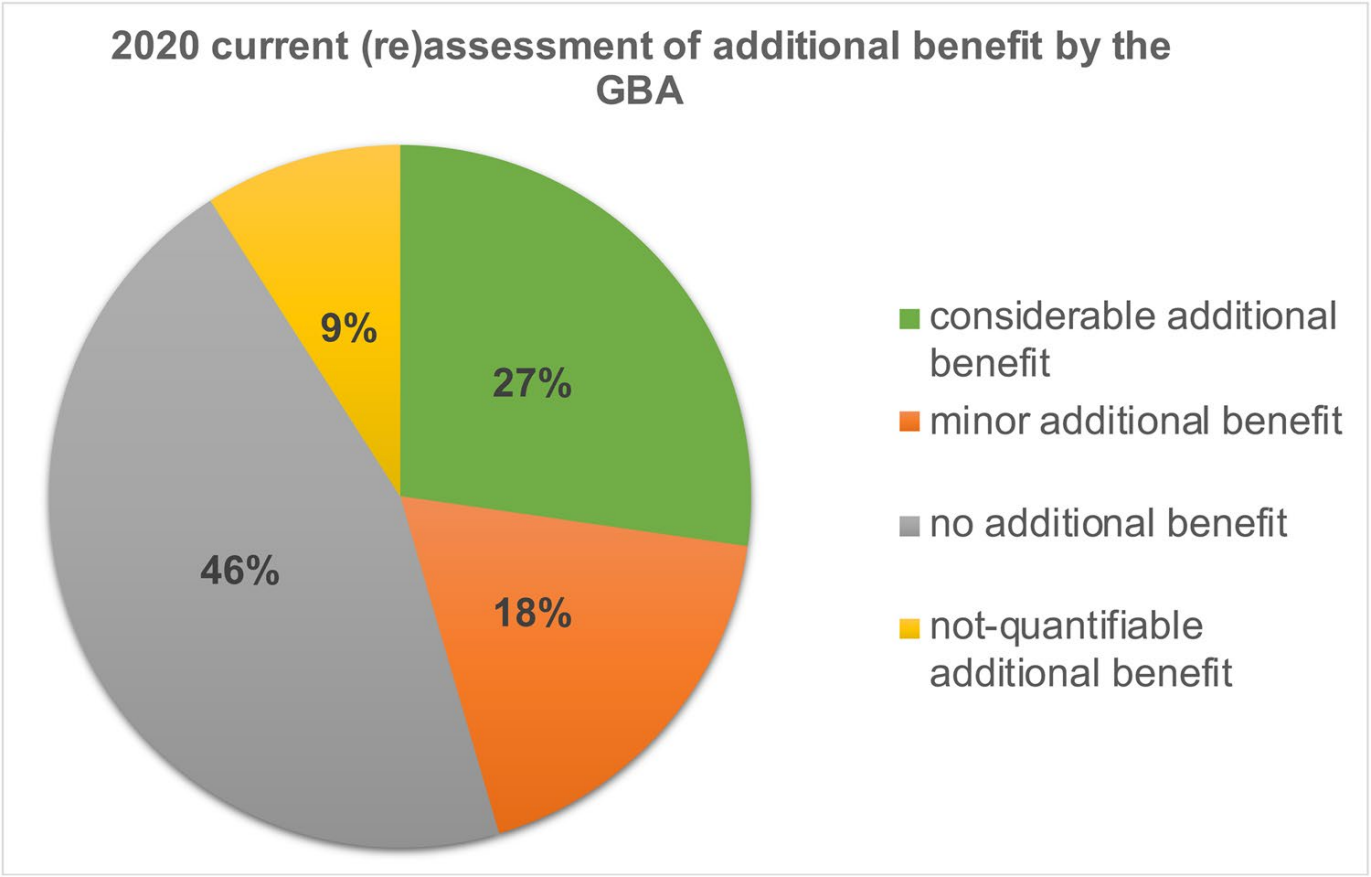
2020 current (re)assessment of additional benefit by the GBA

In the 2020 current (re)assessment by the GBA, no additional benefit could be identified for 46% of the 20 drugs in 33 indications. Eighteen percent had a minor additional benefit, 27% a considerable additional benefit, and for 9%, the additional benefit could not be quantified (Fig. 4).

### Development of benefit assessments in general

Looking at the development of all 20 drugs, it can be seen that between the initial and the current additional benefit assessment 2020, the proportion of drugs without additional benefit decreases by 6%. The share of drugs with a minor additional benefit increases by 3%. Likewise, the proportion of drugs with a considerable additional benefit increases by 6%. The proportion of drugs whose additional benefit is not quantifiable decreases by 3%.

### Development of the Top 4 drugs

**Fig. 5:**
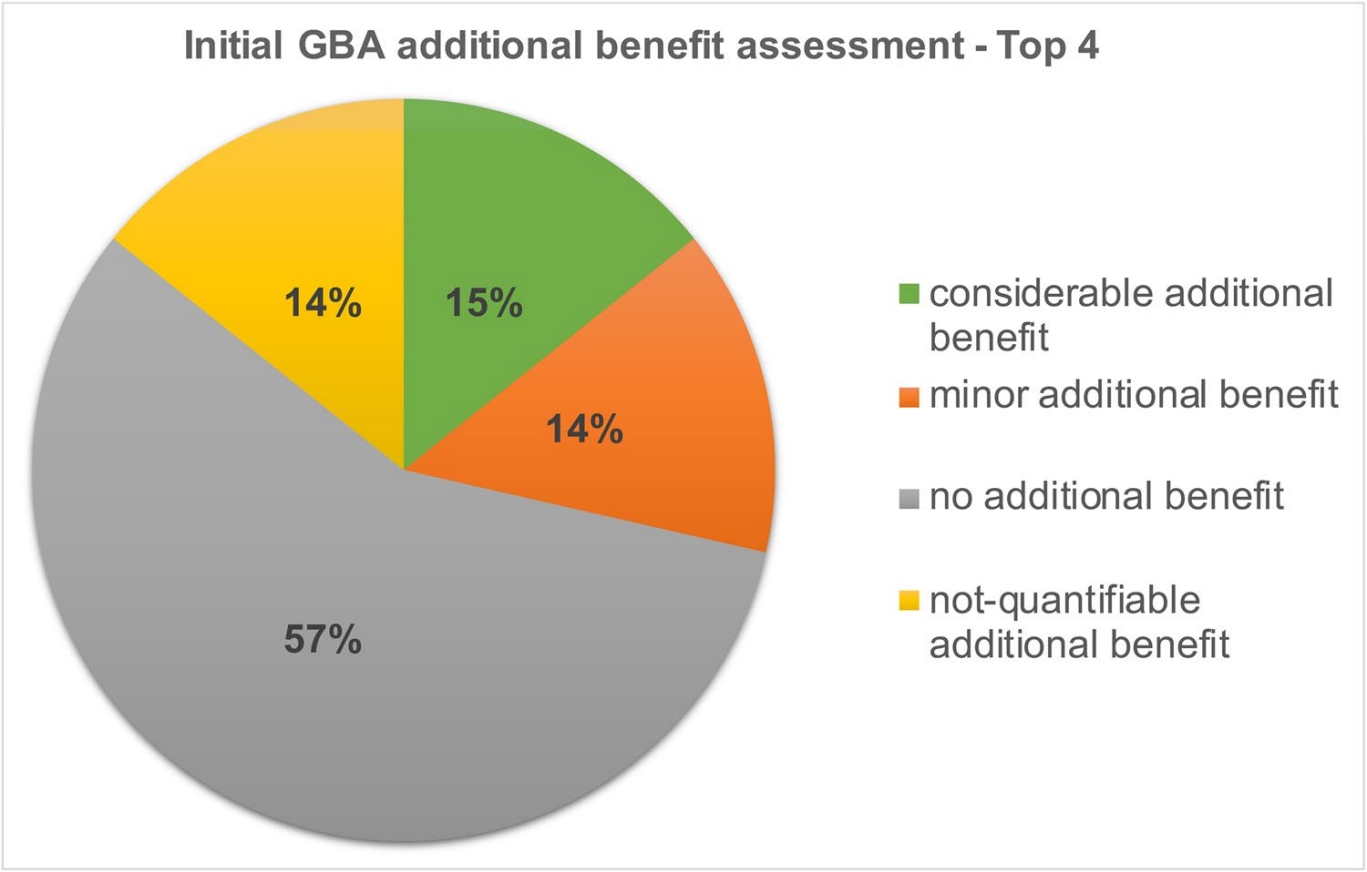
Initial GBA additional benefit assessment—Top 4

**Fig. 6:**
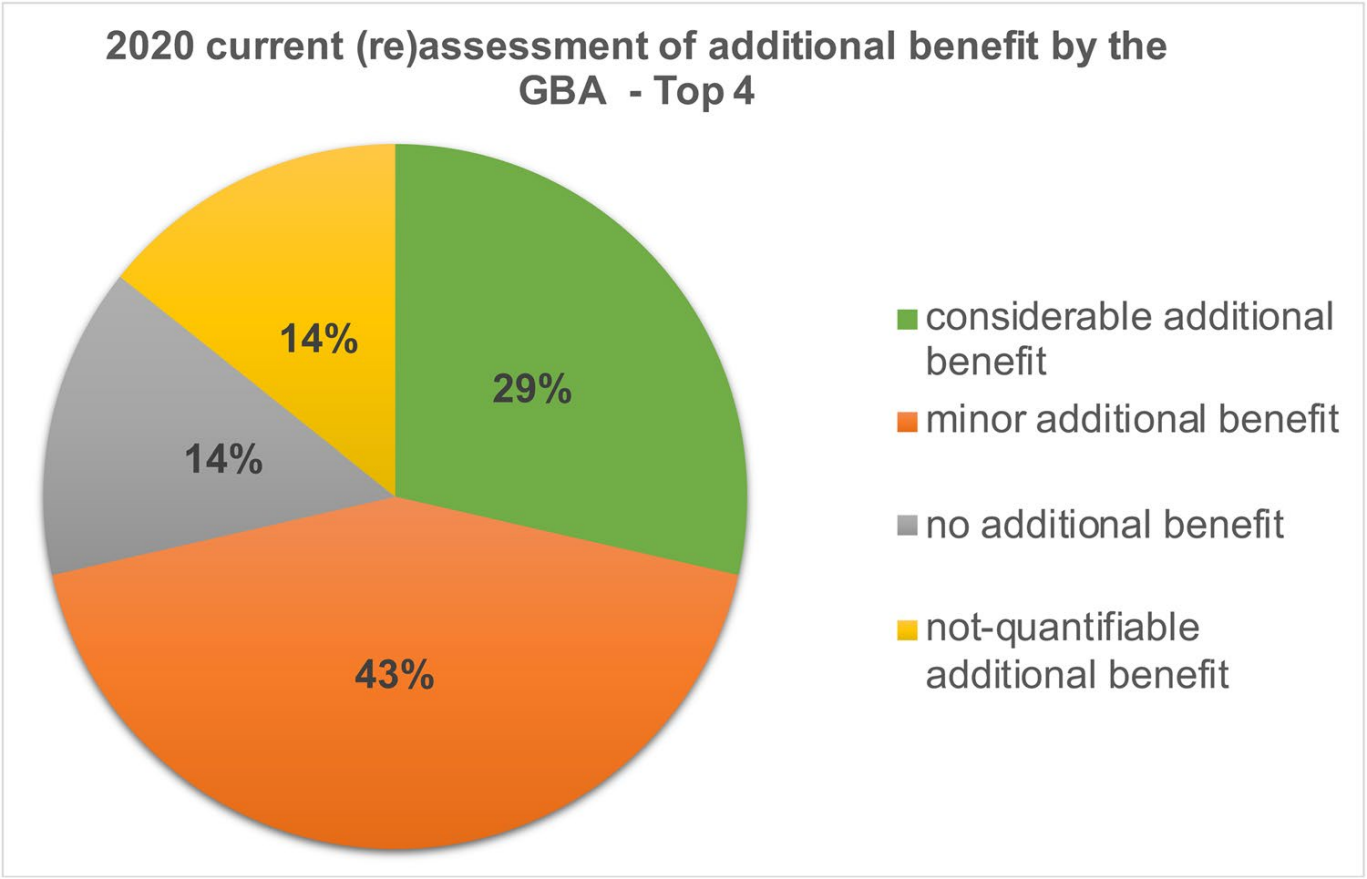
2020 current (re)assessment of additional benefit by the GBA—Top 4

In the initial additional benefit assessment by the GBA, no additional benefit could be identified for 57% of the *Top 4* drugs in 7 indications. Fourteen percent had a minor additional benefit, 15% had a considerable additional benefit, and for another 14%, the additional benefit could not be quantified (Fig. 5). In the 2020 current (re)assessment by the GBA, no additional benefit could be identified for 14% of the *Top 4* drugs. The proportion of drugs without added benefit thus decreased by 43%. For 43% of the drugs, a minor additional benefit could be identified which corresponds to the increase of 29% compared to the initial assessment. The proportion of drugs with considerable added benefit increased by 14 to 29% and the proportion of drugs whose added benefit cannot be quantified remained at 14% (Fig. 6).

### Development of the Flop 5 drugs

**Fig. 7:**
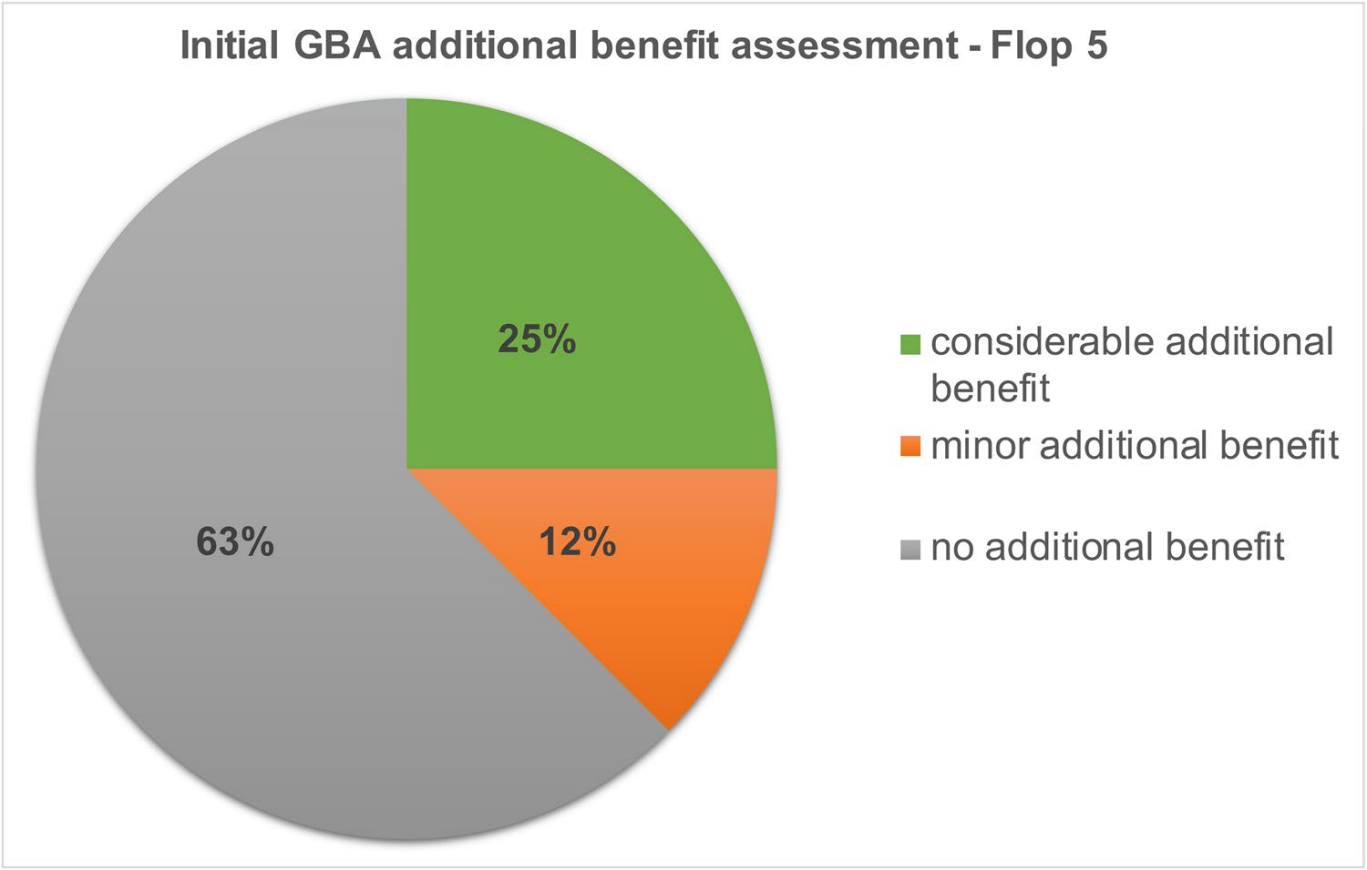
Initial GBA additional benefit assessment—Flop 5

**Fig. 8:**
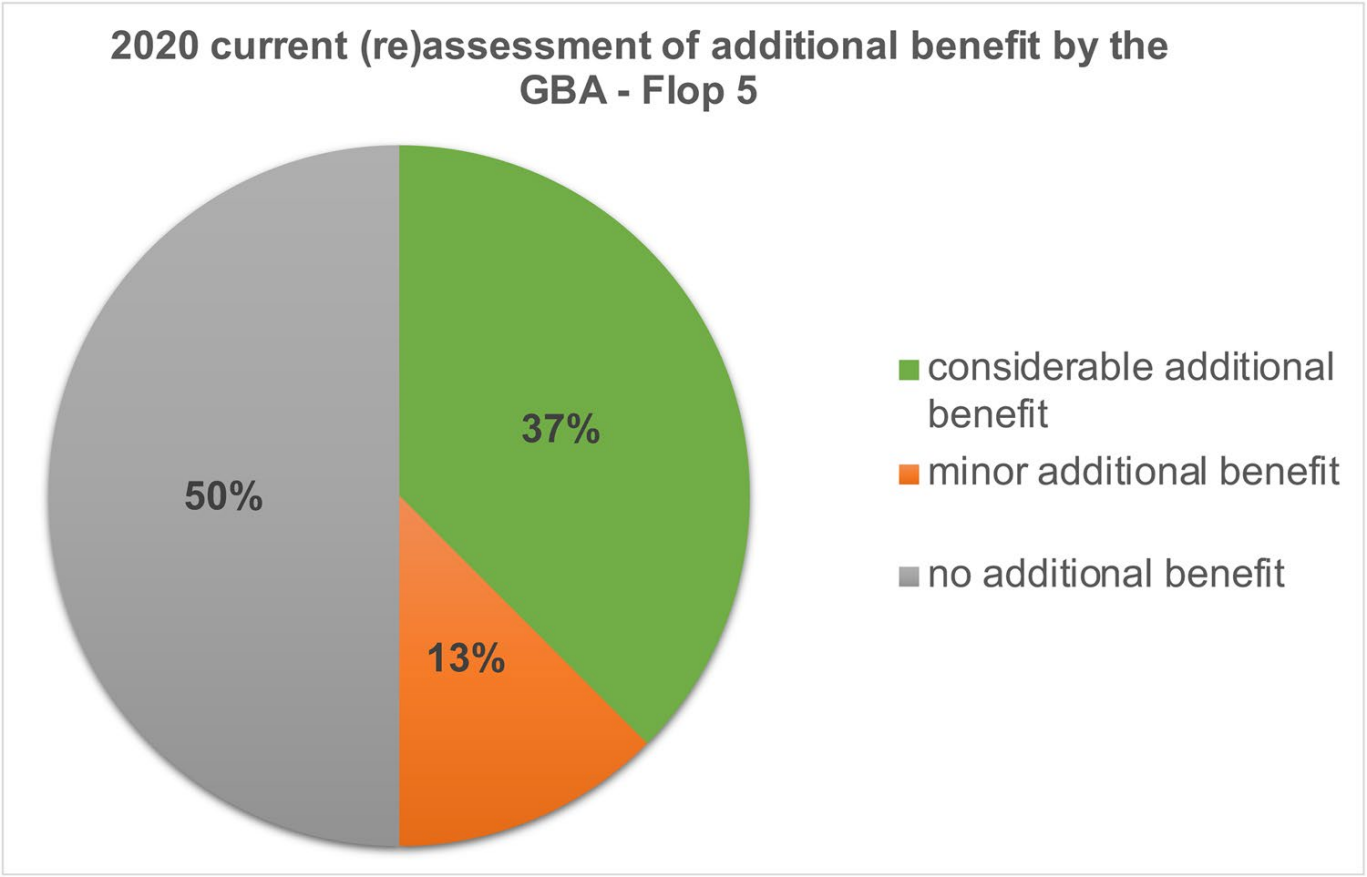
2020 current (re)assessment of additional benefit by the GBA—Flop 5

In the initial additional benefit assessment by the GBA, no additional benefit was identified for 63% of the *Flop 5* drugs in 8 indications. 12% of the drugs had a minor additional benefit and 25% had a considerable additional benefit (Fig. 7). In the 2020 current (re)assessment by the GBA, the proportion of drugs with no added benefit decreased by 13%, corresponding to 50%. The share of drugs with a minor additional benefit increased by 1 to 13%. The proportion of drugs with considerable additional benefit also increased by 12 to 37% (Fig. 8).

### Drugs with no additional benefit

The share of drugs with no additional benefit was initially higher in the group of the *Top 4* as well as in the group of the *Flop 5* than in the total of the 20 drugs considered, whereas the share in the group of the *Flop 5* was higher than in the group of the *Top 4* (57% vs. 52% (value *Top 4* vs. value total) and 63% vs. 52% (value *Flop 5* vs. value total)). In 2020, the proportion of drugs with no additional benefit in the *Top 4* group was considerably lower than in the overall group, whereas the proportion in the *Flop 5* group was higher compared to the overall group (14% vs. 46% and 50% vs. 46%).

### Drugs with not quantifiable additional benefit

In the group of the *Flop 5*, the additional benefit assessment “not quantifiable additional benefit” was not assigned, neither initially nor in 2020. In the *Top 4* group, the proportion of drugs with not quantifiable additional benefit was higher than the overall proportion both initially and in 2020 (14% vs. 12% and 14% vs. 9%).

### Drugs with a minor additional benefit

Looking at the drugs that are assessed with a minor additional benefit, their share was initially lower in the group of the *Top 4* as well as in the group of the *Flop 5* than in the total of the 20 drugs considered, whereby the share in the group of the *Top 4* was higher than in the group of the *Flop 5* (14% vs. 15% and 12% vs. 15%). In 2020, the proportion of drugs with a minor additional benefit in the *Top 4* group was considerably higher than in the overall group, whereas the proportion in the *Flop 5* group was slightly lower than (43% vs. 18% and 13% vs. 18%).

### Drugs with a considerable additional benefit

Initially, the proportion of drugs assessed as having considerable additional benefit in the *Top 4* group was lower than in the overall group, whereas the proportion in the *Flop 5* group was higher (15% vs. 21% and 25% vs. 21%). In 2020, more drugs in both groups were assessed as having considerable additional benefit than in the overall group, but the proportion in the *Flop 5* group was considerably higher than in the *Top 4* group (29% vs. 27% and 37% vs. 27%).

### Evaluation by the ESMO

**Fig. 9:**
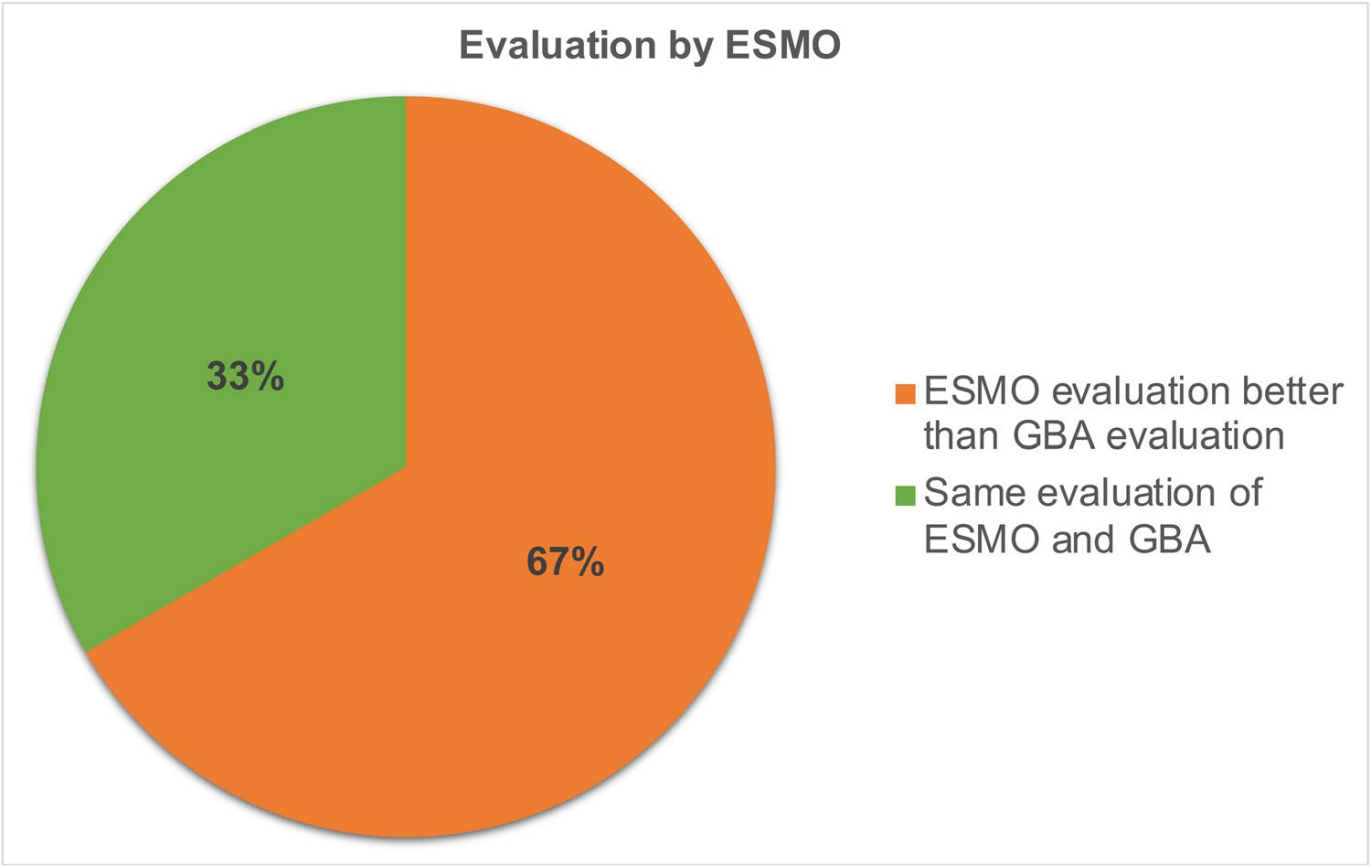
Similarities and differences in the evaluation of Top 4 drugs by GBA and ESMO

ESMO evaluates drugs according to the *ESMO Magnitude of Clinical Benefit Scale* (ESMO-MCBS). The score distinguishes ratings for the palliative and curative settings. In the palliative setting, scores of 1 to 5 can be achieved, with scores of 5 and 4 rated as substantial benefit. In the curative setting, a score of A to C is assigned. Here, scores of A and B are considered substantial benefit. ESMO explicitly states that a high ESMO-MCBS score does not automatically imply a high clinical value of a drug but rather serves as an initial assessment of a drug which must be followed by further investigations in order to use available resources wisely and responsibly (https://www.esmo.org/guidelines/esmo-mcbs/about-the-esmo-mcbs, last accessed November 14, 2022). Based on this score, an assessment is available for 3 of the *Top 4* drugs. Until October 15, 2022, there was no score for nintedanib by the ESMO-MCBS. When comparing the ESMO-MCBS and the GBA evaluation, the ESMO deviates from the GBA evaluation in 4 of 6 evaluations whereby the evaluation of the drug by the ESMO is always considered to be better than by the GBA (Fig. 9).

### Evaluation by the DGHO

**Fig. 10:**
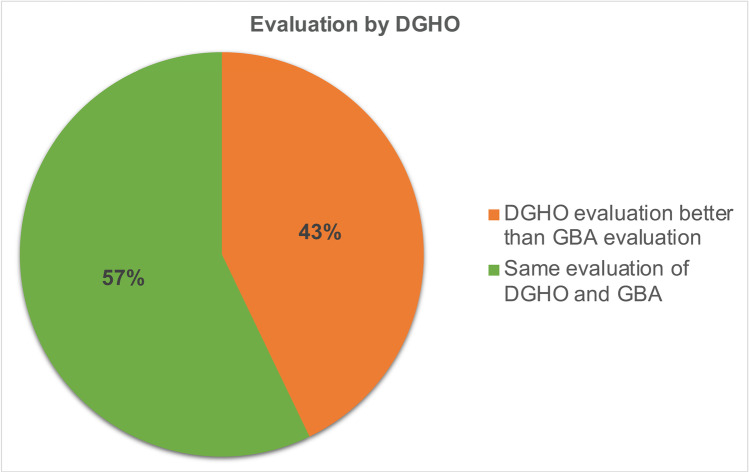
Similarities and differences in the evaluation of Top 4 drugs by GBA and DGHO

The DGHO does not perform a categorical/quantitative evaluation but expresses its opinion on the benefit of a drug in a differentiated written statement. In most cases, the DGHO evaluates the clinical benefit of a drug according to the ESMO-MCBS. In 4 of 7 assessments, the DGHO agrees with the assessment by the GBA, although this comparison is only possible to a limited extent due to the lack of a categorical assessment by the DGHO (Fig. 10). In the statement, the DGHO also deals with the IQWiG report on which the GBA assessment is based. A frequent point of criticism by the DGHO regarding IQWiG reports is a lack of participation by patient and medical expert advisors.

### Advertisements

**Fig. 11:**
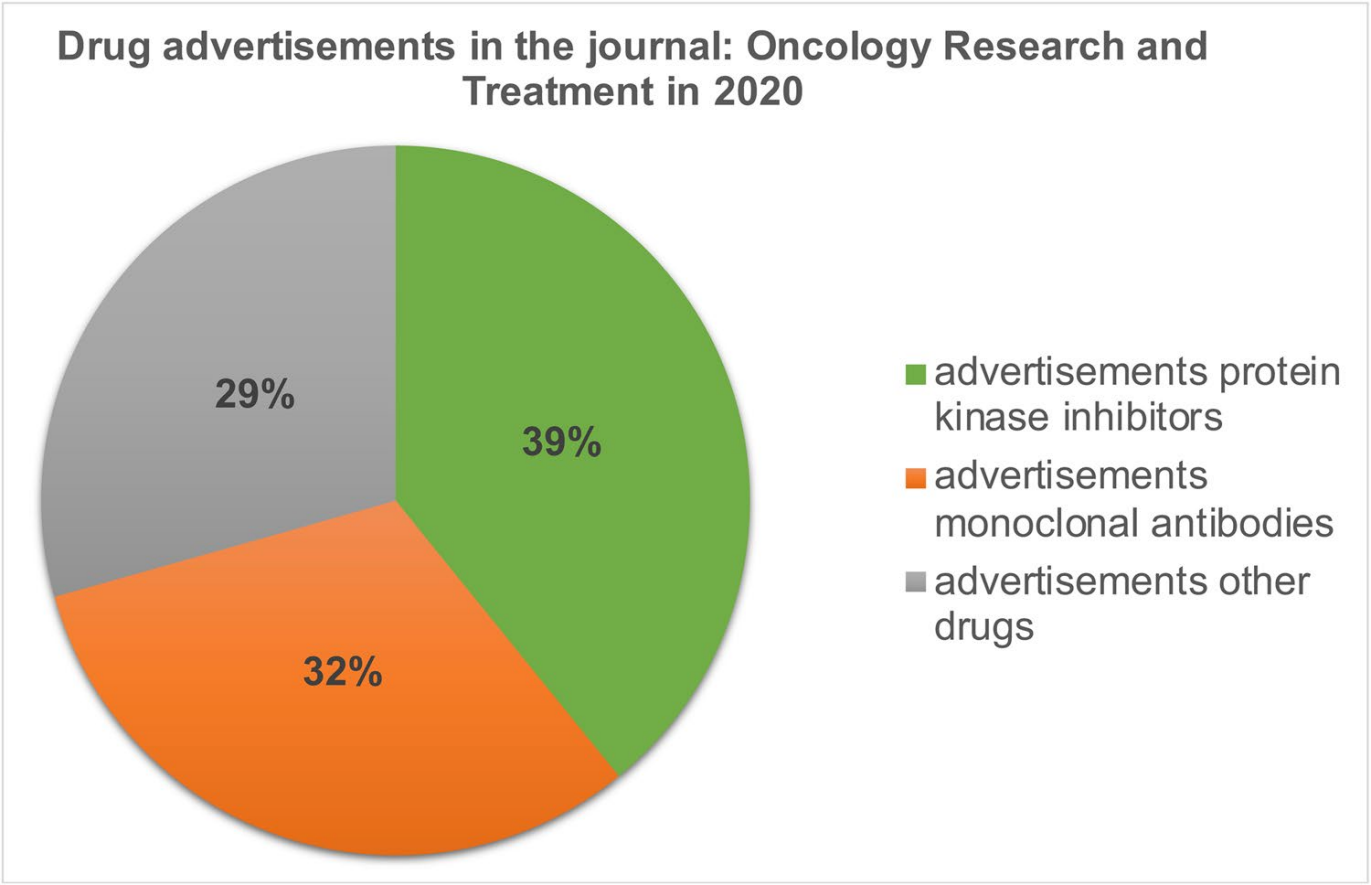
Relative share of drug groups in advertisements

**Fig. 12:**
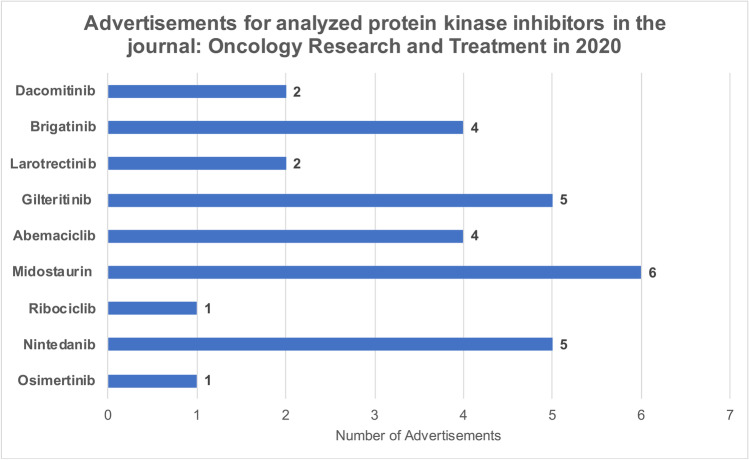
Absolute number of advertisements per drug in 2020

A total of 143 drug advertisements were published in the oncological journal *Oncology Research and Treatment* in 2020. Thirty-nine percent of the advertisements promoted protein kinase inhibitors, 32% monoclonal antibodies, and 29% other drugs (Fig. 11). In 2020, 30 advertisements promoted 9 of the 20 drugs considered in our analysis (Fig. 12). Forty-four percent of these 30 advertisements promoted drugs belonging to the *Flop 5* group and 23% of the 30 advertisements promoted drugs belonging to the *Top 4* group. 57% of the drugs advertised in the 30 advertisements were approved before December 31, 2018, and 43% were approved after December 31, 2018.

## Discussion

### Prescriptions and DDD

The increasing number of prescriptions and DDD show that protein kinase inhibitors are gaining more and more importance for the therapy of oncological diseases due to the increasing change away from classical cytostatics towards new oncologicals, i.e. “targeted therapeutics,” in the sense of precision medicine (Ludwig et al. 2021). Particularly noteworthy in this context is the drug palbociclib, which more than doubles the other *Top 4* drugs in terms of both prescriptions and DDD.

### Sales

The rising sales also illustrate the increasing importance of protein kinase inhibitors in clinical practice. Sales of the two ALK inhibitors brigatinib and ceritinib declined, in contrast to all other drugs considered in this analysis. This can be explained by the availability of two additional ALK inhibitors for the treatment of ALK-positive non-small cell lung cancer (NSCLC) through the drug alectinib, launched in Germany in 2017, and the drug lorlatinib, launched in 2019. However, direct comparative studies between the different ALK inhibitors are still lacking (as of November 21, 2022). A systematic review from 2020 shows that progression-free survival (PFS) was significantly better when treated with alectinib or brigatinib than with therapy with the first-generation ALK inhibitor crizotinib or ceritinib. Likewise, overall survival (OS) seems to improve only with alectinib, compared to chemotherapy and crizotinib, although confounding cannot be precluded here by the authors (Elliott et al. 2020). In another systematic review from 2022, alectinib showed lower toxicity than crizotinib, ceritinib, brigatinib, and lorlatinib, and chemotherapy (Jiang et al. 2022). Nevertheless, the 2020 current additional benefit assessments for ceritinib and brigatinib in individual indications were considerably better than those for alectinib and lorlatinib. This shows that the high drug prices of the protein kinase inhibitors create economic incentives for the pharmaceutical companies (pC), which lead to more me-too drugs being introduced to market instead of developing drugs with new mechanisms of action (Schröder et al. 2021).

### Additional benefit assessment by the GBA

For the drugs considered in our analysis, the GBA did not assign the best rating of “major additional benefit” in any of its evaluations. It evaluates the drugs with a considerable, minor, not quantifiable, or no additional benefit. It can be seen that in the initial evaluation by the GBA, with 52%, no additional benefit could be determined in more than half of the additional benefit evaluations considered. In the 2020 current evaluation by the GBA, with 46%, no additional benefit could be determined in almost half of the cases. These results are consistent with those of an analysis of the DGHO and the *Arbeitsgemeinschaft der wissenschaftlichen medizinischen Fachgesellschaften* (AWMF, *Association of the Scientific Medical Societies*) which considered all additional benefit assessments of the GBA from 2011 to 2020. Nevertheless, it was found that the additional benefit assessments of the drug group of oncology drugs as a whole on average were better than those of the entire drug market. These results raise the question of how valid the additional benefit assessments by the GBA are when often no additional benefit is found, although the respective drugs were approved on the basis of high-quality randomized controlled trials (RCT) (see further discussion in section: Further additional benefit assessments) (Deutsche Gesellschaft für Hämatologie und Onkologie, (DGHO) and Arbeitsgemeinschaft der Wissenschaftlichen Medizinischen Fachgesellschaften e.V., (AWMF), 2021).

### Development of the additional benefit assessments

Looking at the development between the initial additional benefit assessment and the current additional benefit assessment in 2020, it can be seen that the additional benefit assessments are improving overall: the share of evaluations in which no additional benefit can be found or no additional benefit can be quantified is decreasing, whereas the share of evaluations with a minor or considerable additional benefit is increasing. This development can also be seen in the same way for the in *Top 4* group and the *Flop 5* group. When a drug is launched there is often no long-term data available, so that often no or only a minor additional benefit can be found. Only subsequent evaluations show the actual extent of the additional benefit of a drug (Haserück et al. 2022). The only exception is that the proportion of not quantifiable drugs in the group of the *Top 4* remains the same.

### Drugs with no additional benefit

The share of drugs with no additional benefit was higher in the group of the *Flop 5* than in the group of the *Top 4*, both initially and in 2020. It seems to be tolerable that for the drugs with the lowest share of prescriptions, sales, and DDD no additional benefit can be found by the GBA rather than for the drugs with the highest share of prescriptions, sales, and DDD.

### Drugs with not quantifiable additional benefit

According to the rules of procedure of the GBA, in the case of a not quantifiable additional benefit there is an additional benefit, but it is not quantifiable because of lack of scientific data (Gemeinsamer Bundesausschuss 2022). This means that for more than 10% of the drugs with the largest share of prescriptions, sales, and DDD in our analysis, no statement on the added benefit can be made due to insufficient scientific data. This raises the question on which basis these drugs are prescribed so frequently and why they generate such high sales if the GBA cannot make any statement about additional benefit due to insufficient scientific data.

### Drugs with a minor additional benefit

The proportion of drugs with a minor additional benefit was higher in the *Top 4* group than in the *Flop 5* group, both initially and in 2020. It seems coherent that drugs with a larger share of prescriptions and sales are rated better than those with a smaller share. Nevertheless, the *Top 4* drugs are only rated with a minor additional benefit in most cases. According to the high number of prescriptions and sales, a considerable additional benefit would rather be expected for the *Top 4* drugs.

### Drugs with a considerable additional benefit

The share of drugs that are assessed as having considerable additional benefit was higher in the group of the *Flop 5* than in the group of the *Top 4*, both initially and in 2020. Thus, drugs with the lowest share of prescriptions, sales, and DDD are assessed better than drugs with the highest share of prescriptions, sales, and DDD. In this context, prescription numbers, sales, DDD, and additional benefit assessment bear no relation to one another. This paradox raises the question on which basis these drugs are prescribed so frequently and why they generate such high sales if the GBA cannot find any outstanding additional clinical benefit. Likewise, the actual significance of the GBA additional benefit assessment for the clinical practice must be questioned if there is no correlation between the additional benefit assessment and the number of prescriptions, sales, and DDD.

### The case of palbociclib

The lack of correlation between prescriptions and costs on the one hand and the additional benefit assessment by the GBA on the other hand is particularly evident in the example of palbociclib: with 101.64 thousand prescriptions, 247.09 million euros in sales, and 2457.70 thousand DDD in 2020, palbociclib outperforms all other drugs in this analysis. In contrast, no additional benefit was identified in the initial additional benefit assessment by the GBA in 2017 as well as in the reassessment after the deadline in 2019. The pC often justifies the high costs of the oncology drugs by the high expenditures for research and development of these drugs. In fact, however, the costs for research and development are significantly lower than claimed by the company (Schröder et al. 2021). In many cases, the high costs of oncology drugs can therefore neither be justified by high expenditures for research and development nor by any outstanding additional clinical benefit. Comparing the additional benefit assessments of the drug palbociclib by the EMSO and the DGHO with those by the GBA, both ESMO and DGHO identified a substantial benefit, whereas the GBA found no additional benefit in its evaluations. The additional benefit assessments by the two oncological expert associations are therefore exactly the opposite and significantly better than those by the GBA. The reason for this may be a different assessment of the relevance of different endpoints by the different institutions (see section: Further additional benefit assessments). The high number of prescriptions and sales of palbociclib suggest that in practice, clinicians are more oriented towards the additional benefit assessment by ESMO or DGHO. This result questions the clinical significance of the additional benefit assessment by the GBA (see previous section).

### The case of nintedanib

Nintedanib is an angiokinase inhibitor that was approved by the EMA in 2014, under the trade name Vargatef^®^, in combination with docetaxel for the treatment of locally advanced, metastatic, or locally relapsed NSCLC with adenocarcinoma histology after first-line chemotherapy. Under the trade name Ofev^®^, nintedanib was approved in 2015 for the treatment of idiopathic pulmonary fibrosis. This approval has been expanded in 2020 to include therapy for chronic, progressive fibrosing interstitial lung disease (ILDs) and interstitial lung disease in patients with systemic sclerosis (SSc-ILD) (Schwabe and Paffrath 2016). Considering the prescription data for nintedanib, it should be noted that the high prescription numbers and sales figures were primarily achieved by Ofev^®^. Of the 37.37 thousand prescriptions of nintedanib in 2020, 31.5 thousand prescriptions were for Ofev^®^ and 5.8 thousand for Vargatef^®^. Similar trends can be seen in the sales figures: In 2020, nintedanib reached sales of €106.00 million, with €92.2 million in sales from Ofev^®^ and €13.7 million in sales from Vargatef^®^. In the 2020 current additional benefit assessment by the GBA Vargatef^®^ was assessed with a minor additional benefit in the indication NSCLC, whereas Ofev^®^ was assessed with a considerable additional benefit in the indication idiopathic pulmonary fibrosis (Gemeinsamer Bundesausschuss 2015; Gemeinsamer Bundesausschuss 2019). In the indication SSC-ILD, no additional benefit was found for Ofev^®^ in 2020 by the GBA and a minor additional benefit was found for the indication ILDs (Gemeinsamer Bundesausschuss 2021a, Gemeinsamer Bundesausschuss 2021b). Thus, it can be seen that the additional benefit assessments of Ofev^®^ and Vargatef^®^ differ, which limits the meaningfulness of the prescription and sales data for nintedanib considered in this analysis. Nevertheless, this does not reduce the informative value of this analysis as a whole.

### Further additional benefit assessments

The comparison of the additional benefit assessment by the GBA with assessments by the professional societies shows that the different institutions sometimes come to different results. These differences are mainly due to the fact that methodologically different endpoints are recognized as relevant. For example, ESMO assesses the endpoint PFS as relevant in the ESMO-MCBS, whereas the GBA does not do so in most cases in the additional benefit assessments (Deutsche Gesellschaft für Hämatologie und Onkologie, (DGHO) and Arbeitsgemeinschaft der Wissenschaftlichen Medizinischen Fachgesellschaften e.V., (AWMF) 2021). The EMA also evaluates the endpoint PFS in the pivotal studies as relevant. It is therefore incomprehensible why the GBA does not recognize the primary endpoints of the pivotal study, on which the approval of a drug by the EMA is based, as relevant. Methodologically, it is difficult to consider the endpoint OS for oncological diseases with a long survival time (e.g. chronic myeloid leukemia (CML) or metastatic breast cancer (mBC)) separately, since due to the long observation period no clear statement about the effect of the individual drug can be made due to the influence of other drugs. Statements on PFS, on the other hand, are easier to make and run less risk of being influenced by other drugs. Thus, medical evaluation procedures that consider only OS as a relevant endpoint lead to disadvantages for patients (Dabisch et al. 2014). In addition, it must be taken into account that oncological diseases increasingly turn into chronic diseases as a result of therapeutic progress. As a result, the importance of endpoints is changing and PFS is attaining a higher status (Staab et al. 2016). From the patient´s perspective, other endpoints also appear to be gaining in importance; for example, an analysis from 2022 highlights that patients with mBC consider OS to be the most important primary endpoint, but that PFS is also of crucial importance and must therefore be taken into account in medical evaluation procedures as well (Mertz et al. 2022). The differences in results between the institutions are clearer when comparing the European level and the German level (ESMO vs. GBA) than within Germany (DGHO vs. GBA): 67% of the assessments of the *Top 4* drugs by the ESMO differ from the additional benefit assessment by the GBA, whereas only 43% of the assessments of the *Top 4* drugs by the DGHO differ from the additional benefit assessments by the GBA. The reason for this could be a different assessment of the relevance of endpoints at European and German level. According to EU regulation 2021/2282 published in January 2021, the additional benefit assessments of oncologicals are to be the first group of drugs to be evaluated at EU level from 2025. This could be an opportunity to achieve consistent assessments of drugs for clinical practice (Ludwig et al. 2023).

### Advertisements

Advertisements published in the oncological journal *Oncology Research and Treatment* in 2020 primarily promoted "targeted therapeutics", with 71% of advertisements promoting protein kinase inhibitors or monoclonal antibodies. Drugs with the lowest proportion of prescriptions, sales, and DDD in our analysis (*Flop 5*) were advertised almost twice as often as drugs with the highest proportion of prescriptions, sales, and DDD (*Top 4*). The newness of a drug to the market had no effect on the number of advertisements published in this sample. We found no correlation between the number of drug advertisements and the clinical benefit of the drug being promoted. Thus, with the scientific information available to us, there is no scheme why an individual protein kinase inhibitor is promoted or not. Therefore, it should be noted that the high drug costs associated with protein kinase inhibitors attract economic interest among pC to make as much profit as possible (Schröder et al. 2021).

### Limitations of this study

The analyses in this study are based exclusively on publicly available information. Therefore, only apparent discrepancies can be shown. In addition, the prescription, sales, and DDD data refer only to drugs prescribed by physicians for outpatient use and dispensed via public pharmacies at expense of the GKV system. Drugs prescribed via private health insurance and drugs prescribed in hospital are therefore not included in this analysis.

## Conclusions and further perspectives

Our analysis shows that the indication area of a drug develops continuously after its approval. In the following indications, a drug can achieve a better additional benefit assessment by the GBA than in the initial indication. This can be seen as an indirect clinical benefit, as a drug would not be placed on the market due to a negative early benefit assessment by the GBA (in the sense of no additional benefit proven), it would also not be available for other indications in which it might have a considerable additional benefit. The re-evaluations of the additional benefit after expiry of the deadline by the GBA can result in both an improvement and a deterioration of the evaluation. This makes a constant critical examination of the added benefit of a drug absolutely necessary—even after its approval. Particularly considering the immense cost of protein kinase inhibitors, this needs to be well established in clinical practice.

Furthermore, it was shown that the share of a drug in prescriptions, sales, and DDD does not correlate with the clinical benefit of the drug as measured by the corresponding additional benefit assessment by the GBA. The immense costs of oncology drugs are therefore largely caused by drugs for which no additional benefit has been proven by the GBA. This is not only a German phenomenon. In the USA, England, France, and Switzerland, there is also no statistically significant association between monthly therapy costs and clinical benefit of a drug (Vokinger et al. 2020, 2021). Internationally, the systems according to which an additional benefit assessment is carried out are different. For example, in the UK, a direct cost–benefit assessment is performed according to QALYS, but pharmaceutical spending is also raising inexorably in the UK (Rodwin 2021). It remains to be seen whether a uniform EU assessment for oncology drugs will be able to halt this development, especially if, in accordance with EU regulation 2021/2282, the additional benefit is assessed at European level, but pricing continues to take place at national level. In this context, it will be important to pay greater attention to the additional benefit assessment of a drug when setting prices and to adjust the price accordingly (Ludwig et al. 2023).

The examination of other additional benefit assessments underlines that oncological expert associations sometimes deviate from the additional benefit assessment by the GBA. This heterogeneity in the additional benefit assessment is mainly due to the fact that the different institutions consider different endpoints in the studies used for the additional benefit assessment to be clinically relevant. In clinical practice, this makes it difficult to establish a controlled and informed decision for or against a drug. Improved cooperation between the GBA and the professional societies is needed to create consistent assessments in order to achieve clarity for clinical practice. In addition, due to the increasing variability of oncological diseases, e.g. by molecular subtyping or tumor-specific drugs, the relevant endpoints in medical evaluation procedures must also be chosen variably (Dabisch et al. 2014).

The significance of “targeted therapeutics” and especially protein kinase inhibitors can also be seen in the advertisements in scientific journals. The largest share of advertisements promotes “targeted therapeutics,” i.e., drugs that achieve high sales with only low prescription numbers. Here, the economic importance of the new oncology drugs and the related economic interests of the pC become clear. The high profits that pC expect from protein kinase inhibitors lead to an increased number of me-too drugs being brought to the market instead of developing drugs with genuinely new mechanisms of action.

The immense costs of protein kinase inhibitors are not justified due to a lack of correlation between price and additional benefit. Furthermore, the high drug costs, as often claimed by pC, cannot be justified by high expenditures for research and development. In the long-term, the German healthcare system will certainly not be able to bear these costs, especially with regard to the increasingly aging society and the expected increase in the incidence of oncological diseases requiring treatment. Price-regulating measures are therefore urgently needed, especially for drugs whose additional benefits have not been proven. The GKV-Finanzstabilisierungsgesetz (SHI Financial Stabilization Act) from October 2022 provides a reform of the Arzneimittelmarktneuordnungsgesetz (AMNOG, Pharmaceutical Market Reorganization Act) of 2011 and is therefore a first step in the right direction. In the future, larger discounts will be levied on the price negotiated with the company if no or only a minor additional benefit has been determined by the GBA. In addition, the negotiated reimbursement amount will be valid retroactively from the 7th month, rather than from the 13th month as it is currently the case (Bundestag 2022; Haserück et al. 2022). It remains to be seen to what extent this will ensure long-term financial sustainability or whether more far-reaching reforms will be necessary.

## Supplementary Information

Below is the link to the electronic supplementary material.

Supplementary file1 (DOCX 122 KB)

## Data Availability

All source data for this study are available upon reasonable request from the authors.

## Abbreviations

ALK: Anaplastic lymphoma kinase
AML: Acute myeloid leukemia
AVR: Arzneiverordnungsreport (Drug prescription report)
AWMF: Arbeitsgemeinschaft der wissenschaftlichen medizinischen Fachgesellschaften (Association of the Scientific Medical Societies)
BRAF: B-raf proto-oncogene
DGHO: Deutsche Gesellschaft für Hämatologie und Onkologie (German Society for Hematology and Oncology)
EGFR: Epidermal growth factor receptor
FLT3: Fms-like tyrosine kinase 3
GBA: Gemeinsamer Bundesausschuss (Federal Joint Committee)
GKV: Gesetzliche Krankenversicherung (Statutory health insurance, SHI)
HER2: Human epidermal growth factor receptor-2
IQWiG: Institut für Qualität und Wirtschaftlichkeit im Gesundheitswesen (Institute for Quality and Efficiency in Health Care)
NSCLC: Non-small cell lung cancer
WIdO: Wissenschaftliches Institut der Ortskrankenkassen (AOK) (Scientific Institute of the General Local Health Insurance Fund, AOK)
