# Supplementary material for: Critical analysis of the prescription and evaluation of protein kinase inhibitors for oncology in Germany
Source: Naunyn Schmiedebergs Arch Pharmacol. 2023 Apr 4;396(10):2529–43. doi: 10.1007/s00210-023-02475-9 (PMC10497443; doi:10.1007/s00210-023-02475-9)
Supplement: Supplementary file 1 — Supplementary file1 (DOCX 122 KB) [file 210_2023_2475_MOESM1_ESM.docx]

**Supplemental tables S1-S3**

**Caecilia Sophia Obst and Roland Seifert**

**Critical analysis of the prescription and evaluation of protein kinase inhibitors for oncology in Germany**

|  | **Drug, Launch (trading name)** | **Indication** | **Mechanism of Action** | **Prescriptions in year of launch in thousand** | **Prescriptions in 2020 in thousand** | **Change in %** | **Sales in year of launch in € million** | **Sales in 2020 in € million** | **Change in %** | **DDD in years of launch in thousand** | **DDD in 2020 in thousand** | **Change in %** | **DDD-costs in year of launch in €** | **DDD-costs in 2020 in €** | **Change in %** |
| --- | --- | --- | --- | --- | --- | --- | --- | --- | --- | --- | --- | --- | --- | --- | --- |
| **1.** | Ceritinib,  **2015** (Zykadia®) | ALK-positive, advanced NSCLC | ALK inhibitor | 0.30 | 0.40 | + 33.33 | 2.44 | 2.20 | - 9.84 | 13.60 | 12.10 | - 11.03 | 169.53 | 181.36 | + 6.98 |
| **2.** | Cobimetinib, **2015** (Cotellic®) | Metastatic melanoma with BRAF-V600-mutation | MEK inhibitor | 0.10 | 0.82 | + 720.00 | 0.41 | 4.70 | +  1 046.34 | 1.60 | 22.90 | +  1 331.25 | 241.00 | 193.57 | - 19.68 |
| **3.** | Lenvatinib, **2015** (Lenvima®) | Metastatic thyroid carcinoma | Multiple kinase inhibitor | 0.90 | 18.88 | +  1 997.78 | 2.48 | 31.28 | +  1 161.29 | 12.60 | 187.50 | +  1 388.10 | 185.60 | 157.35 | - 15.22 |
| **4.** | Nintedanib, **2015** (Vargatef®) | Metastatic NSCLC | Angiokinase inhibitor | 8.10 | 37.37 | + 361.36 | 26.88 | 106.00 | + 294.35 | 216.10 | 990.50 | + 358.35 | 117.30 | 106.94 | - 8.83 |
| **5.** | Trametinib, **2015** (Mekinist®) | Melanoma with BRAF-V600-mutation | MEK inhibitor | 1.00 | 17.32 | +  1 632.00 | 8.89 | 58.37 | + 556.58 | 29.10 | 405.10 | +  1 292.10 | 288.10 | 144.02 | - 50.01 |
| **6.** | Osimertinib,  **2016** (Tagrisso®) | Metastatic NSCLC with T790M-EGFR-mutation | EGFR inhibitor | 1.50 | 24.65 | +  1 543.30 | 13.18 | 149.72 | +  1 035.96 | 44.90 | 676.00 | +  1 405.57 | 276.81 | 208.72 | - 24.60 |
| **7.** | Palbociclib, **2016** (Ibrance®) | Hormone receptor-positive, HER2-negative locally advanced or metastatic breast cancer | CDK inhibitor | 1.00 | 101.64 | +  10 064.00 | 5.54 | 247.09 | +  4 360.11 | 27.70 | 2 457.70 | +  8 772.56 | 188.78 | 94.79 | - 49.79 |
| **8.**  **Table S 1 Development of protein kinase inhibitors after launch - prescriptions, sales, DDD and DDD-costs** | Alectinib, **2017** (Alecensa®) | ALK-positive, advanced NSCLC, previously treated with crizotinib | ALK inhibitor | 1.10 | 8.51 | + 673.64 | 9.11 | 50.19 | + 450.93 | 30.30 | 238.30 | + 686.47 | 283.50 | 198.48 | - 29.99 |
| **9.** | Midostaurin, **2017** (Rydapt®) | AML with FLT3-mutation; aggressive systemic mastocytosis, systemic mastocytosis with associated haematological neoplasm or mast cell leukaemia | Multiple kinase inhibitor | 0.10 | 2.11 | +  2 010.00 | 2.26 | 33.26 | +  1 371.68 | 2.90 | 59.00 | +  1 934.48 | 735.24 | 531.23 | - 27.75 |
| **10.** | Ribociclib, **2017** (Kisqali®) | Hormone receptor-positive, HER2-negative locally advanced or metastatic breast cancer | CDK inhibitor | 0.50 | 24.11 | +  4 722.00 | 2.85 | 68.80 | +  2 314.04 | 13.10 | 743.50 | +  5 575.57 | 204.85 | 87.23 | - 57.42 |
| **11.** | Tivozanib, **2017** (Fotivda®) | Advanced renal cell carcinoma, first-line treatment | VEGFR inhibitor | 0.03 | 1.43 | +  4 666.67 | 0.14 | 5.60 | +  3 900.00 | 0.80 | 35.30 | +  4 312.50 | 166.13 | 149.53 | - 9.99 |
| **12.** | Abemaciclib, **2018** (Verzenios®) | Hormone receptor-positive, HER2-negative locally advanced or metastatic breast cancer | CDK inhibitor | 0.10 | 9.77 | +  9 670.00 | 0.39 | 24.37 | +  6 148.72 | 2.90 | 218.70 | +  7 441.38 | 126.38 | 105.10 | - 16.84 |
| **13.** | Binimetinib, **2018** (Mektovi®) | Melanoma with BRAF-V600-mutation | MEK inhibitor | 0.30 | 4.50 | +  1 400.00 | 0.91 | 12.34 | +  1 256.04 | 3.60 | 111.90 | +  3 008.33 | 239.42 | 103.92 | - 56.60 |
| **14.** | Encorafenib, **2018** (Braftovi®) | Melanoma with BRAF-V600-mutation | BRAF inhibitor | 0.50 | 5.60 | +  1 020.00 | 0.86 | 30.18 | +  3 409.30 | 3.30 | 123.10 | +  3 630.30 | 245.97 | 231.03 | - 6.07 |
| **15.**  **Table S 1 (continued)** | Brigatinib, **2019** (Alunbrig®) | ALK-positive, advanced NSCLC, previously treated with crizotinib | ALK inhibitor | 0.70 | 0.89 | + 27.14 | 4.05 | 3.47 | - 14.32 | 13.60 | 17.60 | + 29.41 | 281.02 | 185.70 | - 33.92 |
| **16.** | Dacomitinib, **2019** (Vizimpro®) | NSCLC with EGFR-activation mutations, first-line treatment | EGFR inhibitor | 0.03 | 0.20 | + 566.67 | 0.13 | 0.58 | + 346.15 | 0.60 | 4.40 | + 633.33 | 202.83 | 129.20 | - 36.30 |
| **17.** | Gilteritinib, **2019** (Xospata®) | AML with FLT3-mutation | FLT3 inhibitor | 0.02 | 0.51 | +  2 450.00 | 0.43 | 11.33 | +  2 534.88 | 0.50 | 14.30 | +  2 760.00 | 805.60 | 751.71 | - 6.69 |
| **18.** | Larotrectinib, **2019** (Vitrakvi®) | Tumors that display a Neurotrophic Tyrosine Receptor Kinase gene fusion | TRK inhibitor | 0.10 | 0.59 | + 490.00 | 0.40 | 3.91 | + 877.50 | 0.60 | 6.30 | + 950.00 | 633.83 | 584.87 | - 7.72 |
| **19.** | Lorlatinib, **2019** (Lorviqua®) | ALK-positive, advanced NSCLC | ALK inhibitor | 0.40 | 1.30 | + 225.00 | 3.41 | 8.42 | + 146.92 | 12.40 | 37.00 | + 198.39 | 258.98 | 214.76 | - 17.07 |
| **20.** | Neratinib, **2019** (Nerlynx®) | Hormone receptor positive, HER2-overexpressed/ amplified breast cancer | HER2 inhibitor | 0.00 | 0.68 | + 68.00 | 0.03 | 4.04 | +  13 366.67 | 0.10 | 20.40 | +  20 300.00 | 246.00 | 186.48 | - 24.20 |

**Table S 1 (continued)**

|  | **Drug  (trading name)** | **Launch, Indication** | **First GBA benefit assessment** | **Studies** | **GBA reassessment** | **Studies** | **Further Studies** |
| --- | --- | --- | --- | --- | --- | --- | --- |
| **1a.** | Ceritinib (Zykadia®) | **2015:** ALK-positive, advanced NSCLC, previously treated with crizotinib | **2015:** No additional benefit [1], [2] | A2201 [3], X2101 [4] | **2017:** considerable additional benefit [5], [6] | A2303 (ASCEND-5) [7] | [142], [143], ASCEND-8 [144] |
| **1b.** | Ceritinib (Zykadia®) | **2017:** ALK-positive, advanced NSCLC, first-line treatment | **2017**: No additional benefit [8], [9] | A2301 (ASCEND-4) [10], PROFILE 1014 [11], PROFILE 1029 [12] |  |  | ASCEND-8 [144] |
| **2.** | Cobimetinib (Cotellic®) | **2015:** Metastatic melanoma with BRAF-V600-mutation | **2016:** Considerable additional benefit [13], [14] | GO28141 (coBRIM) [15], [16] |  |  | coBRIM Follow-Up [17] |
| **3a.** | Lenvatinib (Lenvima®) | **2015:** Metastatic thyroid carcinoma | **2015:** Not-quantifiable additional benefit, orphan drug [18], [19] | SELECT [20] | **2019:** no additional benefit [21], [22] | SELECT [20), DECISION [23] | [145], [146] |
| **3b.** | Lenvatinib (Lenvima®) | **2016:** Advanced renal cell carcinoma | **2016:** Minor additional benefit [24], [25] | E7080-G000-205 [26] | **2021:** no additional benefit [27], [28] | E7080-G000-205 [26], METEOR [29] | CLEAR [147], [148] |
| **3c.** | Lenvatinib  (Lenvima®) | **2018:** Advanced or unresectable hepatocellular carcinoma | **2018:** No additional benefit [30], [31] | REFLECT [32] |  |  | [149] |
| **3d.** | Lenvatinib  (Lenvima®) | **2021:** Endometrial carcinoma, previously treated with platinum-containing therapy, in combination with pembrolizumab | **As at January 21th 2022:** Start of process: 15.12.2021, decision-making estimated early in July 2022 |  |  |  |  |
| **3e.** | Lenvatinib (Lenvima®) | **2021:** Advanced renal cell carcinoma, first-line treatment, in combination with pembrolizumab | **As at February 2th 2022:** Start of process: 15.12.2021, decision-making estimated early July 2022 |  |  |  |  |
| **4.** | Nintedanib  (Vargatef®) | **2015:** Metastatic NSCLC | **2015:** Minor additional benefit [33], [34] | LUME-Lung1 [35] |  |  | [150], [151] |
| **5a.**  **Table S 2 Development of protein kinase inhibitors after launch - first GBA benefit assessment and reassessment** | Trametinib (Mekinist®) | **2015:** Melanoma with BRAF-V600-mutation, in combination with dabrafenib | **2016:** Considerable additional benefit [36], [37] | COMBI-v [38] |  |  | [39] |
| **5b.** | Trametinib (Mekinist®) | **2017:**  Advanced NSCLC with BRAF-V600-mutation, in combination with dabrafenib | **2017:** No additional benefit [40], [41] | BRF113928 [42], Cardella 2013 [43], Ding 2017 [44], Registeranalyse NGM Köln 2017 [45] |  |  | [152] |
| **6a.** | Osimertinib (Tagrisso®) | **2016:** Metastatic NSCLC with T790M-EGFR-mutation | **2016:** No additional benefit [46], [47] | AURAex [48], AURA2 [49], IMPRESS [50] | **2017:** Considerable additional benefit [51], [52] | AURA3 [53] | [153] |
| **6b.** | Osimertinib  (Tagrisso®) | **2019:** Metastatic NSCLC with T790M-EGFR-mutation, first-line treatment | **2019:** Considerable additional benefit [54], [55] | FLAURA [56] |  |  | [153] |
| **6c.** | Osimertinib (Tagrisso®) | **2021:** Metastatic NSCLC with T790M-EGFR-mutation, adjuvant treatment | **2021:** Not-quantifiable additional benefit [57], [58] | ADAURA [59] |  |  |  |
| **7.** | Palbociclib (Ibrance®) | **2016:** Hormone receptor-positive, HER2-negative, locally advanced or metastatic breast cancer | **2017:** No additional benefit [60], [61] | PALOMA-1 [62], PALOMA-2 [63], PALOMA-3 [64] | **2019:** No additional benefit [65], [66] | PALOMA-3 [64] | [154] |
| **8a.** | Alectinib (Alecensa®) | **2017:** ALK-positive, advanced NSCLC, previously treated with crizotinib | **2017:** Minor additional benefit [67], [68], [69] | ALUR [70] |  |  | [155], [156] |
| **8b.** | Alectinib  (Alecensa®) | **2017:** ALK-positive, advanced NSCLC, first-line treatment | **2018:** Not-quantifiable additional benefit [71], [72] | ALEX [73] |  |  | [157] |
| **9a.** | Midostaurin (Rydapt®) | **2017:** AML with FLT3-mutation | **2018:** Considerable additional benefit, orphan drug [74], [75] | RATIFY [76] |  |  | [158] |
| **9b.**  **Table S 2 (continued)** | Midostaurin (Rydapt®) | **2017:** Aggressive systemic mastocytosis, systemic mastocytosis with associated haematological neoplasm or mast cell leukaemia | **2018:** Not-quantifiable additional benefit [77], [78] | CPKC412D2201 [79], CPKC412A2213 [80] |  |  | [159], [160] |
| **10a.** | Ribociclib (Kisqali®) | **2017:** Hormone receptor-positive, HER2-negative, locally advanced or metastatic breast cancer, in combination with an aromatase inhibitor | **2018:** No additional benefit [81], [82] | MONALEESA-2 [83] | **2020:** Minor additional benefit [84], [85] | MONALEESA-2 [86] | [161], MONALEESA-7 [162] |
| **10b.** | Ribociclib (Kisqali®) | **2017:** Hormone receptor-positive, HER2-negative, locally advanced or metastatic breast cancer, in combination with fulvestrant | **2019:** No additional benefit [87], [88] | MONALEESA-3 [89] | **2020:** Minor additional benefit [90], [91] | MONALEESA-3 [92] | [163] |
| **11.** | Tivozanib (Fotivda®) | **2017:** Advanced renal cell carcinoma, first-line treatment | **2018:** No additional benefit [93], [94] | TIVO-1 [95], SWITCH [96],  ASSURE [97] |  |  | [164], TIVO-3 [165] |
| **12a.** | Abemaciclib  (Verzenios®) | **2018:** Hormone receptor-positive, HER2-negative, locally advanced or metastatic breast cancer, in combination with an aromatase inhibitor | **2019:** No additional benefit [98], [99] | MONARCH-3 [100] |  |  | [166], [167] |
| **12b.** | Abemaciclib (Verzenios®) | **2018:** Hormone receptor-positive, HER2-negative, locally advanced or metastatic breast cancer, in combination with fulvestrant | **2019**: No additional benefit [101], [102] | MONARCH-2 [103] | **2020:** No additional benefit [104], [105] | MONARCH-2 [106], MONARCHplus [107] | [168] |
| **13.** | Binimetinib (Mektovi®) | **2018:** Melanoma with BRAF-V600-mutation, in combination with encorafenib | **2019**: No additional benefit [108], [109] | COLUMBUS [110], coBRIM [16] |  |  | [169] |
| **14a.**  **Table S 2 (continued)** | Encorafenib (Braftovi®) | **2018:** Melanoma with BRAF-V600-mutation, in combination with binimetinib | **2019:** No additional benefit [111], [112] | COLUMBUS [110], coBRIM [16] |  |  | [169] |
| **14b.** | Encorafenib (Braftovi®) | **2020:** Metastatic colorectal cancer with BRAF-V600-mutation after prior systemic therapy, in combination with cetuximab | **2020:** Considerable additional benefit [113], [114] | BEACON CRC [115] |  |  | [170] |
| **15a.** | Brigatinib (Alunbrig®) | **2019:** ALK-positive, advanced NSCLC, previously treated with crizotinib | **2019:** No additional benefit [116], [117] | ALTA [118], ASCEND-5 [7], AP26113-11-101 [119], ALTA-1L [120] |  |  | [171] |
| **15b.** | Brigatinib (Alunbrig®) | **2020:** ALK-positive, advanced NSCLC, previously not treated with an ALK inhibitor, with brain metastases | **2020:** Considerable additional benefit [121], [122] | ALTA-1L [120] |  |  | [172] |
| **15c.** | Brigatinib (Alunbrig®) | **2020:** ALK-positive, advanced NSCLC, previously not treated with an ALK inhibitor, without brain metastases | **2020:** Minor additional benefit [121], [122] | ALTA-1L [120] |  |  | [172] |
| **16.** | Dacomitinib (Vizimpro®) | **2019:** NSCLC with EGFR-activating mutations, first-line treatment | **2019:** No additional benefit [123], [124] | ARCHER 1050 [125], [126] |  |  | [173] |
| **17.** | Gilteritinib (Xospata®) | **2019:** AML with FLT3-mutation | **2020:** Considerable additional benefit, orphan drug [127], [128] | ADMIRAL [129], CHRYSALIS [130] |  |  | [174] |
| **18.** | Larotrectinib (Vitrakvi®) | **2019:** Tumors that display a Neurotrophic Tyrosine Receptor Kinase gene fusion | **2020:** No additional benefit [131], [132] | LOXO-TRK-14001 [133], NAVIGATE [134], SCOUT [135] |  |  | [175] |
| **19.** | Lorlatinib (Lorviqua®) | **2019:** ALK-positive, advanced NSCLC | **2019:** No additional benefit [136], [137] | B7461001 [138] |  |  | [176], [177] |
| **20.** | Neratinib (Nerlynx®) | **2019:** Hormone receptor- positive, HER2-overexpressed/amplified breast cancer | **2020:** Minor additional benefit [139], [140] | ExteNET [141] |  |  | [178], [179] |

**Table S 2 (continued)**

|  | **Drug (trading name)** | **Mechanism of Action** | **Target** | **Action** | **pIC50** |
| --- | --- | --- | --- | --- | --- |
| **1.** | Ceritinib (Zykadia^®^) | ALK inhibitor | ALK | Inhibition | 9.7 |
|  |  |  | Insulin receptor | Inhibition | 8.1 |
|  |  |  | IGF1 | Inhibition | 8.1 |
|  |  |  | FLT3 | Inhibition | 7.2 |
|  |  |  | TSSK1B | Inhibition | 7.6 |
| **2.** | Cobimetinib (Cotellic^®^) | MEK inhibitor | MEK1 | Negative allosteric modulation | 9.1 |
|  |  |  | MEK2 | Negative allosteric modulation | 6.7 |
|  |  |  | MEK7 | Negative allosteric modulation | <5.0 |
| **3.** | Lenvatinib (Lenvima^®^) | Multiple kinase inhibitor | VEGFR-2 | Inhibition | 8.4 |
|  |  |  | VEGFR-3 | Inhibition | 8.3 |
| **4.**  **Table S 3 Pharmacological characterization of analyzed drugs** | Nintedanib (Vargatef^®^) | Angiokinase inhibitor | VEGFR-3 | Inhibition | 7.9 |
|  |  |  | VEGFR-2 | Inhibition | 7.7 |
|  |  |  | VEGFR-1 | Inhibition | 7.5 |
|  |  |  | FGFR2 | Inhibition | 7.4 |
|  |  |  | PDGFR α | Inhibition | 7.2 |
|  |  |  | PDGFR β | Inhibition | 7.2 |
|  |  |  | FGFR1 | Inhibition | 7.2 |
|  |  |  | FGFR3 | Inhibition | 7.0 |
|  |  |  | FGFR4 | Inhibition | 6.2 |
| **5.** | Trametinib (Mekinist^®^) | MEK inhibitor | MEK1 | Inhibition | 9.0-9.1 |
|  |  |  | MEK2 | Inhibition | 8.7 |
| **6.** | Osimertinib (Tagrisso^®^) | EGFR inhibitor | EGFR | Inhibition | 6.3 |
| **7.** | Palbociclib (Ibrance^®^) | CDK inhibitor | CDK4 | Inhibition | 7.4-8.5 |
|  |  |  | CDK6 | Inhibition | 7.4-8.0 |
| **8.** | Alectinib (Alecensa^®^) | ALK inhibitor | ALK | Inhibition | 8.7 |
| **9.** | Midostaurin (Rydapt^®^) | Multiple kinase inhibitor | FLT3 | Inhibition | 6.3 |
| **10.** | Ribociclib (Kisqali^®^) | CDK inhibitor | CDK4 | Inhibition | 8.0 |
| **11.** | Tivozanib (Fotivda^®^) | VEGFR inhibitor | VEGFR-2 | Inhibition | 9.8 |
|  |  |  | VEGFR-1 | Inhibition | 9.7 |
|  |  |  | VEGFR-3 | Inhibition | 9.6 |
| **12.** | Abemaciclib (Verzenios^®^) | CDK inhibitor | CDK4 | Inhibition | 8.7 |
|  |  |  | CDK6 | Inhibition | 8.0 |
| **13.**  **Table S 3 (continued)** | Binimetinib (Mektovi^®^) | MEK inhibitor | MEK1 | Negative allosteric modulation | 7.9 |
|  |  |  | MEK2 | Negative allosteric modulation | 7.9 |
| **14.** | Encorafenib (Braftovi^®^) | BRAF inhibitor | BRAF-V600 | Inhibition | 8.4 |
| **15.** | Brigatinib (Alunbrig^®^) | ALK inhibitor | ALK | Inhibition | 9.2-9.4 |
|  |  |  | IGF1R | Inhibition | 7.6 |
|  |  |  | EGFR | Inhibition | 6.9 |
|  |  |  | Insulin receptor | Inhibition | 6.7 |
| **16.** | Dacomitinib (Vizimpro^®^) | EGFR inhibitor | EGFR | Inhibition | 8.2 |
|  |  |  | HER2/neu | Inhibition | 7.3 |
|  |  |  | ERBB4 | Inhibition | 7.1 |
| **17.** | Gilteritinib (Xospata^®^) | FLT3 inhibitor | LTK | Inhibition | 9.7 |
|  |  |  | FLT3 | Inhibition | 9.5 |
|  |  |  | ALK | Inhibition | 9.3 |
|  |  |  | AXL | Inhibition | 9.1 |
|  |  |  | NTRK1 | Inhibition | 9.0 |
|  |  |  | ROS1 | Inhibition | 8.8 |
|  |  |  | Ret proto-oncogene | Inhibition | 8.8 |
|  |  |  | MERTK | Inhibition | 8.5 |
| **18.**  **Table S 3 (continued)** | Larotrectinib (Vitrakvi^®^) | TRK inhibitor | NTRK1 | Inhibition | 8.0 |
| **19.** | Lorlatinib (Lorviqua^®^) | ALK inhibitor | FES | Inhibition | 8.2 |
|  |  |  | ROS1 | Inhibition | 11.3 (pKi) |
|  |  |  | ALK | Inhibition | 9.1 (pKi) |
| **20.** | Neratinib (Nerlynx^®^) | HER2 inhibitor | HER2/neu | Inhibition | 7.2 |
|  |  |  | EGFR | Inhibition | 7.0 |

**Table S 3 (continued)**

ALK ALK receptor tyrosine kinase

AXL AXL receptor tyrosine kinase

BRAF-V600 BRAF^V600E^ kinase

CDK4 cyclin dependent kinase 4

CDK6 cyclin dependent kinase 6

EGFR epidermal growth factor receptor

ERBB4 erb-b2 receptor tyrosine kinase 4

FES FES proto-oncogene, receptor tyrosine kinase

FGFR1 fibroblast growth factor receptor 1

FGFR2 fibroblast growth factor receptor 2

FGFR3 fibroblast growth factor receptor 3

FGFR4 fibroblast growth factor receptor 4

FLT3 Fms related receptor tyrosine kinase 3

HER2/neu human epidermal growth factor receptor-2

IGF1 Insulin-like growth factor I receptor

LTK leukocyte receptor tyrosine kinase

MEK1 mitogen-activated protein kinase 1

MEK2 mitogen-activated protein kinase 2

MEK7 mitogen-activated protein kinase 7

MERTK MER proto-oncogene, tyrosine kinase

NTRK1 neurotrophic receptor kinase 1

PDGFR α platelet derived growth factor receptor alpha

PDGFR β platelet derived growth factor receptor beta

ROS1 c-ros oncogene 1, receptor tyrosine kinase

TSSK1B testis specific serine kinase 1B

VEGFR-1 vascular endothelial growth factor receptor 1

VEGFR-2 vascular endothelial growth factor receptor 2

VEGFR-3 vascular endothelial growth factor receptor 3

**Reference Table S 3:** IUPHAR/BPS Guide to Pharmacology: https://www.guidetopharmacology.org. Accessed 16 March 2023

**Reference list Table 2:**

[1]: Institut für Qualität und Wirtschaftlichkeit im Gesundheitswesen (IQWiG) (2015) Ceritinib – Nutzenbewertung gemäß § 35a SGB V. https://www.g-ba.de/downloads/92-975-880/2015-09-29_Nutzenbewertung-IQWiG_Ceritinib.pdf. Accessed 29 January 2022

[2]: Gemeinsamer Bundesausschuss (GBA) (2015) Beschluss des Gemeinsamen Bundesausschusses über eine Änderung der Arzneimittel-Richtlinie (AM-RL): Anlage XII - Beschlüsse über die Nutzenbewertung von Arzneimitteln mit neuen Wirkstoffen nach § 35a SGB V - Ceritinib. https://www.g-ba.de/downloads/39-261-2414/2015-12-17_AM-RL-XII_Ceritinib_2015-07-01-D-171_BAnz.pdf. Accessed 29 January 2022

[3]: Crinò L, Ahn M, De Marinis F, Groen HJM, Wakelee H, Hida T, Mok T, Spigel D, Felip E, Nishio M, Scagliotti G, Branle F, Emeremni C, Quadrigli M, Zhang J, Shaw AT (2016) Multicenter Phase II Study of Whole-Body and Intracranial Activity With Ceritinib in Patients With ALK-Rearranged Non-Small-Cell Lung Cancer Previously Treated With Chemotherapy and Crizotinib: Results From ASCEND-2. J Clin Oncol *34,* 2866-2873. DOI: 10.1200/JCO.2015.65.5936

[4]: Kim D, Mehra R, Tan DSW, Felip E, Chow LQM, Camidge DR, Vansteenkiste J, Sharma S, De Pas T, Riely GJ, Solomon BJ, Wolf J, Thomas M, Schuler M, Liu G, Santoro A, Sutradhar S, Li S, Szczudlo T, Yovine A, Shaw AT (2016) Activity and safety of ceritinib in patients with ALK-rearranged non-small-cell lung cancer (ASCEND-1): updated results from the multicentre, open-label, phase 1 trial. Lancet Oncol *17,* 452-463. DOI: 10.1016/S1470-2045(15)00614-2

[5]: Institut für Qualität und Wirtschaftlichkeit im Gesundheitswesen (IQWiG) (2017) Ceritinib (Nicht kleinzelliges Lungenkarzinom) - Nutzenbewertung gemäß § 35a SGB V (Ablauf Befristung). https://www.g-ba.de/downloads/92-975-1688/2017-01-02_Nutzenbewertung- IQWiG_Ceritinib-2016-10-01-D-259.pdf. Accessed 29 January 2022

[6]: Gemeinsamer Bundesausschuss (GBA) (2017) Beschluss des Gemeinsamen Bundesausschusses über eine Änderung der Arzneimittel-Richtlinie (AM-RL): Anlage XII - Beschlüsse über die Nutzenbewertung von Arzneimitteln mit neuen Wirkstoffen nach § 35a SGB V - Ceritinib (Ablauf der Befristung). https://www.g-ba.de/downloads/39-261-2876/2017-03-16_AM-RL-XII_Ceritinib_D-259_BAnz.pdf. Accessed 29 January 2022

[7]: Shaw AT, Kim TM, Crinò L, Gridelli C, Kiura K, Liu G, Novello S, Bearz A, Gautschi O, Mok T, Nishio M, Scagliotti G, Spigel DR, Deudon S, Zheng C, Pantano S, Urban P, Massacesi C, Viraswami-Appanna K, Felip E (2017) Ceritinib versus chemotherapy in patients with ALK-rearranged non-small-cell lung cancer previously given chemotherapy and crizotinib (ASCEND-5): a randomised, controlled, open-label, phase 3 trial. Lancet Oncol *18,* 874-886. DOI: 10.1016/S1470-2045(17)30339-X

[8]: Institut für Qualität und Wirtschaftlichkeit im Gesundheitswesen (IQWiG) (2017) Ceritinib (nicht kleinzelliges Lungenkarzinom) - Nutzenbewertung gemäß § 35a SGB V. https://www.g-ba.de/downloads/92-975-2007/2017-08-01_Nutzenbewertung-IQWiG_Ceritinib-D-296.pdf. Accessed 29 January 2022

[9]: Gemeinsamer Bundesausschuss (GBA) (2018) Beschluss des Gemeinsamen Bundesausschusses über eine Änderung der Arzneimittel-Richtlinie (AM-RL): Anlage XII - Beschlüsse über die Nutzenbewertung von Arzneimitteln mit neuen Wirkstoffen nach § 35a SGB V - Ceritinib (neues Anwendungsgebiet: Erstlinienbehandlung, nicht-kleinzelliges Lungenkarzinom). https://www.g-ba.de/downloads/39-261-3214/2018-02-01_AM-RL-XII_Ceritinib_nAWG_D-296_BAnz.pdf. Accessed 29 January 2022

[10]: Soria J, Tan DSW, Chiari R, Wu Y, Paz-Ares L, Wolf J, Geater SL, Orlov S, Cortinovis D, Yu C, Hochmair M, Cortot AB, Tsai C, Moro-Sibilot D, Campelo RG, McCulloch T, Sen P, Dugan M, Pantano S, Branle F, Massacesi C, de Castro G (2017) First-line ceritinib versus platinum-based chemotherapy in advanced ALK-rearranged non-small-cell lung cancer (ASCEND-4): a randomised, open-label, phase 3 study. Lancet *389,* 917-929. DOI: 10.1016/S0140-6736(17)30123-X

[11]: Solomon BJ, Mok T, Kim D, Wu Y, Nakagawa K, Mekhail T, Felip E, Cappuzzo F, Paolini J, Usari T, Iyer S, Reisman A, Wilner KD, Tursi J, Blackhall F (2014) First-line crizotinib versus chemotherapy in ALK-positive lung cancer. N Engl J Med *371,* 2167-2177. DOI: 10.1056/NEJMoa1408440

[12]: Wu Y, Lu S, Lu Y, Zhou J, Shi Y, Sriuranpong V, Ho JCM, Ong CK, Tsai C, Chung C, Wilner KD, Tang Y, Masters ET, Selaru P, Mok TS (2018) Results of PROFILE 1029, a Phase III Comparison of First-Line Crizotinib versus Chemotherapy in East Asian Patients with ALK-Positive Advanced Non-Small Cell Lung Cancer. J Thorac Oncol *13,* 1539-1548. DOI: 10.1016/j.jtho.2018.06.012

[13]: Institut für Qualität und Wirtschaftlichkeit im Gesundheitswesen (IQWiG) (2016) Cobimetinib - Nutzenbewertung gemäß § 35a SGB V. https://www.g-ba.de/downloads/92-975-1254/2016-03-11_A15-52_Cobimetinib_Nutzenbewertung-35a-SGB-V.pdf. Accessed 29 January 2022

[14]: Gemeinsamer Bundesausschuss (GBA) (2016) Beschluss des Gemeinsamen Bundesausschusses über eine Änderung der Arzneimittel-Richtlinie (AM-RL): Anlage XII - Beschlüsse über die Nutzenbewertung von Arzneimitteln mit neuen Wirkstoffen nach § 35a SGB V - Cobimetinib. https://www.g-ba.de/downloads/39-261-2607/2016-06-02_AM-RL-XII_Cobimetinib_D-196_BAnz.pdf. Accessed 29 January 2022

[15]: Larkin J, Ascierto PA, Dréno B, Atkinson V, Liszkay G, Maio M, Mandalà M, Demidov L, Stroyakovskiy D, Thomas L, de la Cruz-Merino L, Dutriaux C, Garbe C, Sovak MA, Chang I, Choong N, Hack SP, McArthur GA, Ribas A (2014) Combined vemurafenib and cobimetinib in BRAF-mutated melanoma. N Engl J Med *371,* 1867-1876. DOI: 10.1056/NEJMoa1408868

[16]: Ascierto PA, McArthur GA, Dréno B, Atkinson V, Liszkay G, Di Giacomo AM, Mandalà M, Demidov L, Stroyakovskiy D, Thomas L, de la Cruz-Merino L, Dutriaux C, Garbe C, Yan Y, Wongchenko M, Chang I, Hsu JJ, Koralek DO, Rooney I, Ribas A, Larkin J (2016) Cobimetinib combined with vemurafenib in advanced BRAF(V600)-mutant melanoma (coBRIM): updated efficacy results from a randomised, double-blind, phase 3 trial. Lancet Oncol *17,* 1248-1260. DOI: 10.1016/S1470-2045(16)30122-X

[17]: Ascierto PA, Dréno B, Larkin J, Ribas A, Liszkay G, Maio M, Mandalà M, Demidov L, Stroyakovskiy D, Thomas L, de la Cruz-Merino L, Atkinson V, Dutriaux C, Garbe C, Hsu J, Jones S, Li H, McKenna E, Voulgari A, McArthur GA (2021) 5-Year Outcomes with Cobimetinib plus Vemurafenib in BRAFV600 Mutation-Positive Advanced Melanoma: Extended Follow-up of the coBRIM Study. Clin Cancer Res *27,* 5225-5235. DOI: 10.1158/1078-0432.CCR-21-0809

[18]: Gemeinsamer Bundesausschuss (GBA) (2015) Nutzenbewertung von Arzneimittel mit neuen Wirkstoffen nach § 35a SGB V, Bewertung von Arzneimitteln für seltene Leiden nach § 35a Absatz 1 Satz 10 i.V.m. 5. Kapitel § 12 Nr. 1 Satz 2 VerfO, Wirkstoff: Lenvatinib. https://www.gba.de/downloads/92-975-885/2015-10-01_Nutzenbewertung-G-BA_Lenvatinib.pdf. Accessed 29 January 2022

[19]: Gemeinsamer Bundesausschuss (GBA) (2015) Beschluss des Gemeinsamen Bundesausschusses über eine Änderung der Arzneimittel-Richtlinie (AM-RL): Anlage XII - Beschlüsse über die Nutzenbewertung von Arzneimitteln mit neuen Wirkstoffen nach § 35a SGB V - Lenvatinib. https://www.g-ba.de/downloads/39-261-2413/2015-12-17_AM-RL-XII_Lenvatinib_2015-07-01-D-164_BAnz.pdf. Accessed 29 January 2022

[20]: Schlumberger M, Tahara M, Wirth LJ, Robinson B, Brose MS, Elisei R, Habra MA, Newbold K, Shah MH, Hoff AO, Gianoukakis AG, Kiyota N, Taylor MH, Kim S, Krzyzanowska MK, Dutcus CE, de las Heras B, Zhu J, Sherman SI (2015) Lenvatinib versus placebo in radioiodine-refractory thyroid cancer. N Engl J Med *372,* 621-630. DOI: 10.1056/NEJMoa1406470

[21]: Institut für Qualität und Wirtschaftlichkeit im Gesundheitswesen (IQWiG) (2019) Lenvatinib (Schilddrüsenkarzinom) - Nutzenbewertung gemäß § 35a SGB V. https://www.g- ba.de/downloads/92-975-2957/2019-02-15_Nutzenbewertung-IQWiG_Lenvatinib-D-428.pdf. Accessed 29 January 2022

[22]: Gemeinsamer Bundesausschuss (GBA) (2015) Beschluss des Gemeinsamen Bundesausschusses über eine Änderung der Arzneimittel-Richtlinie (AM-RL): Anlage XII - Nutzenbewertung von Arzneimitteln mit neuen Wirkstoffen nach § 35a SGB V Lenvatinib (Bewertung nach Aufhebung des Orphan Drug-Status). https://www.g-ba.de/downloads/39-261-3922/2019-08-15_AM-RL-XII_Lenvatinib_D-428_BAnz.pdf. Accessed 29 January 2022

[23]: Brose MS, Nutting CM, Jarzab B, Elisei R, Siena S, Bastholt L, de la Fouchardiere C, Pacini F, Paschke R, Shong YK, Sherman SI, Smit JWA, Chung J, Kappeler C, Peña C, Molnár I, Schlumberger MJ (2014) Sorafenib in radioactive iodine-refractory, locally advanced or metastatic differentiated thyroid cancer: a randomised, double-blind, phase 3 trial. Lancet *384,* 319-328. DOI: 10.1016/S0140-6736(14)60421-9

[24]: Institut für Qualität und Wirtschaftlichkeit im Gesundheitswesen (IQWiG) (2017) Lenvatinib (Nierenzellkarzinom) - Nutzenbewertung gemäß § 35a SGB V. https://www.g-ba.de/downloads/92-975-1682/2016-10-01_Nutzenbewertung-IQWiG_Lenvatinib-D-257.pdf. Accessed 29 January 2022

[25]: Gemeinsamer Bundesausschuss (GBA) (2017) Beschluss des Gemeinsamen Bundesausschusses über eine Änderung der Arzneimittel-Richtlinie (AM-RL): Anlage XII - Beschlüsse über die Nutzenbewertung von Arzneimitteln mit neuen Wirkstoffen nach § 35a SGB V - Lenvatinib (neues Anwendungsgebiet: fortgeschrittenes Nierenzellkarzinom). https://www.g-ba.de/downloads/39-261-2878/2017-03-16_AM-RL-XII_Lenvatinib_D-257_BAnz.pdf. Accessed 29 January 2022

[26]: Motzer RJ, Hutson TE, Glen H, Michaelson MD, Molina A, Eisen T, Jassem J, Zolnierek J, Maroto JP, Mellado B, Melichar B, Tomasek J, Kremer A, Kim H, Wood K, Dutcus C, Larkin J (2015) Lenvatinib, everolimus, and the combination in patients with metastatic renal cell carcinoma: a randomised, phase 2, open-label, multicentre trial. Lancet Oncol *16,* 1473-1482. DOI: 10.1016/S1470-2045(15)00290-9

[27]: Institut für Qualität und Wirtschaftlichkeit im Gesundheitswesen (IQWiG) (2021) Lenvatinib (Nierenzellkarzinom) - Nutzenbewertung gemäß § 35a SGB V (Ablauf Befristung). https://www.g-ba.de/downloads/92-975-4367/2021-01-01_Nutzenbewertung-IQWiG_Lenvatinib_D-620.pdf. Accessed 29 January 2022

[28]: Gemeinsamer Bundesausschuss (GBA) (2021) Beschluss des Gemeinsamen Bundesausschusses über eine Änderung der Arzneimittel-Richtlinie (AM-RL): Anlage XII - Nutzenbewertung von Arzneimitteln mit neuen Wirkstoffen nach § 35a SGB V, Lenvatinib (Neubewertung nach Fristablauf: fortgeschrittenes Nierenzellkarzinom, Kombination mit Everolimus). https://www.g-ba.de/downloads/39-261-4908/2021-07-01_AM-RL-XII_Lenvatinib_D-620_BAnz.pdf. Accessed 29 January 2022

[29]: Choueiri TK, Escudier B, Powles T, Tannir NM, Mainwaring PN, Rini BI, Hammers HJ, Donskov F, Roth BJ, Peltola K, Lee JL, Heng DYC, Schmidinger M, Agarwal N, Sternberg CN, McDermott DF, Aftab DT, Hessel C, Scheffold C, Schwab G, Hutson TE, Pal S, Motzer RJ (2016) Cabozantinib versus everolimus in advanced renal cell carcinoma (METEOR): final results from a randomised, open-label, phase 3 trial. Lancet Oncol *17,* 917-927. DOI: 10.1016/S1470-2045(16)30107-3

[30]: Institut für Qualität und Wirtschaftlichkeit im Gesundheitswesen (IQWiG) (2019) Lenvatinib (hepatozelluläres Karzinom) - Nutzenbewertung gemäß § 35a SGB V. https://www.g-ba.de/downloads/92-975-2630/2018-10-01_Nutzenbewertung-IQWiG_Lenvatinib-D-379.pdf. Accessed 29 January 2022

[31]: Gemeinsamer Bundesausschuss (GBA) (2019) Beschluss des Gemeinsamen Bundesausschusses über eine Änderung der Arzneimittel-Richtlinie (AM-RL): Anlage XII - Beschlüsse über die Nutzenbewertung von Arzneimitteln mit neuen Wirkstoffen nach § 35a SGB V - Lenvatinib (neues Anwendungsgebiet Hepatozelluläres Karzinom). https://www.g-ba.de/downloads/39-261-3715/2019-03-22_AM-RL-XII_Lenvatinib-nAWG_D-379_BAnz.pdf. Accessed 29 January 2022

[32]: Kudo M, Finn RS, Qin S, Han K, Ikeda K, Piscaglia F, Baron A, Park J, Han G, Jassem J, Blanc JF, Vogel A, Komov D, Evans TRJ, Lopez C, Dutcus C, Guo M, Saito K, Kraljevic S, Tamai T, Ren M, Cheng A (2018) Lenvatinib versus sorafenib in first-line treatment of patients with unresectable hepatocellular carcinoma: a randomised phase 3 non-inferiority trial. Lancet *391,* 1163-1173. DOI: 10.1016/S0140-6736(18)30207-1

[33]: Institut für Qualität und Wirtschaftlichkeit im Gesundheitswesen (IQWiG) (2015) Nintedanib - Nutzenbewertung gemäß § 35a SGB V. https://www.g-ba.de/downloads/92-975-738/2015-03-30_A15-01_Nintedanib_Nutzenbewertung-35a-SGB-V.pdf. Accessed 29 January 2022

[34]: Gemeinsamer Bundesausschuss (GBA) (2015) Beschluss des Gemeinsamen Bundesausschusses über eine Änderung der Arzneimittel-Richtlinie (AM-RL): Anlage XII - Beschlüsse über die Nutzenbewertung von Arzneimitteln mit neuen Wirkstoffen nach § 35a SGB V - Nintedanib. https://www.g-ba.de/downloads/39-261-2262/2015-06-18_AM-RL-XII_Nintedanib_2015-01-01-D-147_BAnz.pdf. Accessed 29 January 2022

[35]: Reck M, Kaiser R, Mellemgaard A, Douillard J, Orlov S, Krzakowski M, von Pawel J, Gottfried M, Bondarenko I, Liao M, Gann C, Barrueco J, Gaschler-Markefski B, Novello S (2014) Docetaxel plus nintedanib versus docetaxel plus placebo in patients with previously treated non-small-cell lung cancer (LUME-Lung 1): a phase 3, double-blind, randomised controlled trial. Lancet Oncol *15,* 143-155. DOI: 10.1016/S1470-2045(13)70586-2

[36]: Institut für Qualität und Wirtschaftlichkeit im Gesundheitswesen (IQWiG) (2016) Trametinib - Nutzenbewertung gemäß § 35a SGB V. https://www.g-ba.de/downloads/92-975-1180/2015-12-28_A15-40_Trametinib_Nutzenbewertung-35a-SGB-V.pdf. Accessed 30 January 2022

[37]: Gemeinsamer Bundesausschuss (GBA) (2016) Beschluss des Gemeinsamen Bundesausschusses über eine Änderung der Arzneimittel-Richtlinie (AM-RL): Anlage XII - Beschlüsse über die Nutzenbewertung von Arzneimitteln mit neuen Wirkstoffen nach § 35a SGB V - Trametinib. https://www.g-ba.de/downloads/39-261-2531/2016-03-17_AM-RL-XII_Trametinib_2015-10-01-D-183_BAnz.pdf. Accessed 30 January 2022

[38]: Grob JJ, Amonkar MM, Karaszewska B, Schachter J, Dummer R, Mackiewicz A, Stroyakovskiy D, Drucis K, Grange F, Chiarion-Sileni V, Rutkowski P, Lichinitser M, Levchenko E, Wolter P, Hauschild A, Long GV, Nathan P, Ribas A, Flaherty K, Sun P, Legos JJ, McDowell DO, Mookerjee B, Schadendorf D, Robert C (2015) Comparison of dabrafenib and trametinib combination therapy with vemurafenib monotherapy on health-related quality of life in patients with unresectable or metastatic cutaneous BRAF Val600-mutation-positive melanoma (COMBI-v): results of a phase 3, open-label, randomised trial. Lancet Oncol *16,* 1389-1398. DOI: 10.1016/S1470-2045(15)00087-X

[39]: Robert C, Grob JJ, Stroyakovskiy D, Karaszewska B, Hauschild A, Levchenko E, Chiarion Sileni V, Schachter J, Garbe C, Bondarenko I, Gogas H, Mandalá M, Haanen JBAG, Lebbé C, Mackiewicz A, Rutkowski P, Nathan PD, Ribas A, Davies MA, Flaherty KT, Burgess P, Tan M, Gasal E, Voi M, Schadendorf D, et al (2019) Five-Year Outcomes with Dabrafenib plus Trametinib in Metastatic Melanoma. N Engl J Med *381,* 626-636. DOI: 10.1056/NEJMoa1904059

[40]: Institut für Qualität und Wirtschaftlichkeit im Gesundheitswesen (IQWiG) (2017) Trametinib (nicht kleinzelliges Lungenkarzinom) - Nutzenbewertung gemäß § 35a SGB V. https://www.g-ba.de/downloads/92-975-1912/2017-05-01_Nutzenbewertung-IQWiG-Trametinib-D-284.pdf. Accessed 30 January 2022

[41]: Gemeinsamer Bundesausschuss (GBA) (2017) Beschluss des Gemeinsamen Bundesausschusses über eine Änderung der Arzneimittel-Richtlinie (AM-RL): Anlage XII - Beschlüsse über die Nutzenbewertung von Arzneimitteln mit neuen Wirkstoffen nach § 35a SGB V - Trametinib (neues Anwendungsgebiet: nicht-kleinzelliges Lungenkarzinom). https://www.g-ba.de/downloads/39-261-3095/2017-10-19_AM-RL-XII_Trametinib_D-284_BAnz.pdf. Accessed 30 January 2022

[42]: Planchard D, Besse B, Groen HJM, Souquet P, Quoix E, Baik CS, Barlesi F, Kim TM, Mazieres J, Novello S, Rigas JR, Upalawanna A, D'Amelio AM, Zhang P, Mookerjee B, Johnson BE (2016) Dabrafenib plus trametinib in patients with previously treated BRAF(V600E)-mutant metastatic non-small cell lung cancer: an open-label, multicentre phase 2 trial. Lancet Oncol *17,* 984-993. DOI: 10.1016/S1470-2045(16)30146-2

[43]: Cardarella S, Ogino A, Nishino M, Butaney M, Shen J, Lydon C, Yeap BY, Sholl LM, Johnson BE, Jänne PA (2013) Clinical, pathologic, and biologic features associated with BRAF mutations in non-small cell lung cancer. Clin Cancer Res *19,* 4532-4540. DOI: 10.1158/1078-0432.CCR-13-0657

[44]: Ding X, Zhang Z, Jiang T, Li X, Zhao C, Su B, Zhou C (2017) Clinicopathologic characteristics and outcomes of Chinese patients with non-small-cell lung cancer and BRAF mutation. Cancer Med *6,* 555-562. DOI: 10.1002/cam4.1014

[45]: Netzwerk Genomische Medizin (2017) Auswertung einer Register-Analyse von Patienten mit fortgeschrittenem NSCLC und BRAF-V600E-Mutation.

[46]: Institut für Qualität und Wirtschaftlichkeit im Gesundheitswesen (IQWiG) (2016) Osimertinib (Lungenkarzinom) - Nutzenbewertung gemäß § 35a SGB V. https://www.g-ba.de/downloads/92-975-1409/A16-14_Osimertinib_Nutzenbewertung-35a-SGB-V.pdf. Accessed 08 February 2022

[47]: Gemeinsamer Bundesausschuss (GBA) (2016) Beschluss des Gemeinsamen Bundesausschusses über eine Änderung der Arzneimittel-Richtlinie (AM-RL): Anlage XII - Beschlüsse über die Nutzenbewertung von Arzneimitteln mit neuen Wirkstoffen nach § 35a SGB V - Osimertinib. https://www.g-ba.de/downloads/39-261-2700/2016-09-15_AM-RL-XII_Osimertinib_D-219_BAnz.pdf. Accessed 08 February 2022

[48]: Yang JC, Ahn M, Kim D, Ramalingam SS, Sequist LV, Su W, Kim S, Kim J, Planchard D, Felip E, Blackhall F, Haggstrom D, Yoh K, Novello S, Gold K, Hirashima T, Lin C, Mann H, Cantarini M, Ghiorghiu S, Jänne PA (2017) Osimertinib in Pretreated T790M-Positive Advanced Non-Small-Cell Lung Cancer: AURA Study Phase II Extension Component. J Clin Oncol *35,* 1288-1296. DOI: 10.1200/JCO.2016.70.3223

[49]: Goss G, Tsai C, Shepherd FA, Bazhenova L, Lee JS, Chang G, Crino L, Satouchi M, Chu Q, Hida T, Han J, Juan O, Dunphy F, Nishio M, Kang J, Majem M, Mann H, Cantarini M, Ghiorghiu S, Mitsudomi T (2016) Osimertinib for pretreated EGFR Thr790Met-positive advanced non-small-cell lung cancer (AURA2): a multicentre, open-label, single-arm, phase 2 study. Lancet Oncol *17,* 1643-1652. DOI: 10.1016/S1470-2045(16)30508-3

[50]: Soria J, Wu Y, Nakagawa K, Kim S, Yang J, Ahn M, Wang J, Yang JC, Lu Y, Atagi S, Ponce S, Lee DH, Liu Y, Yoh K, Zhou J, Shi X, Webster A, Jiang H, Mok TSK (2015) Gefitinib plus chemotherapy versus placebo plus chemotherapy in EGFR-mutation-positive non-small-cell lung cancer after progression on first-line gefitinib (IMPRESS): a phase 3 randomised trial. Lancet Oncol *16,* 990-998. DOI: 10.1016/S1470-2045(15)00121-7

[51]: Institut für Qualität und Wirtschaftlichkeit im Gesundheitswesen (IQWiG) (2017) Osimertinib (nicht kleinzelliges Lungenkarzinom) - Nutzenbewertung gemäß § 35a SGB V (Ablauf Befristung). https://www.g-ba.de/downloads/92-975-1904/2017-08-01_Nutzenbewertung-IQWiG_Osimertinib-D-282.pdf. Accessed 08 February 2022

[52]: Gemeinsamer Bundesausschuss (GBA) (2017) Beschluss des Gemeinsamen Bundesausschusses über eine Änderung der Arzneimittel-Richtlinie (AM-RL): Anlage XII - Beschlüsse über die Nutzenbewertung von Arzneimitteln mit neuen Wirkstoffen nach § 35a SGB V - Osimertinib (Ablauf der Befristung). https://www.g-ba.de/downloads/39-261-3092/2017-10-19_AM-RL-XII_Osimertinib_D-282_BAnz.pdf. Accessed 08 February 2022

[53]: Mok TS, Wu Y, Ahn M, Garassino MC, Kim HR, Ramalingam SS, Shepherd FA, He Y, Akamatsu H, Theelen WSME, Lee CK, Sebastian M, Templeton A, Mann H, Marotti M, Ghiorghiu S, Papadimitrakopoulou VA (2017) Osimertinib or Platinum-Pemetrexed in EGFR T790M-Positive Lung Cancer. N Engl J Med *376,* 629-640. DOI: 10.1056/NEJMoa1612674

[54]: Institut für Qualität und Wirtschaftlichkeit im Gesundheitswesen (IQWiG) (2018) Osimertinib (nicht kleinzelliges Lungenkarzinom) - Nutzenbewertung gemäß § 35a SGB V. https://www.g-ba.de/downloads/92-975-2507/2018-07-15_Nutzenbewertung-IQWiG_Osimertinib-D-369.pdf. Accessed 08 February 2022

[55]: Gemeinsamer Bundesausschuss (GBA) (2019) Beschluss des Gemeinsamen Bundesausschusses über eine Änderung der Arzneimittel-Richtlinie (AM-RL): Anlage XII - Beschlüsse über die Nutzenbewertung von Arzneimitteln mit neuen Wirkstoffen nach § 35a SGB V - Osimertinib (neues Anwendungsgebiet: Erstlinientherapie des lokal fortgeschrittenen oder metastasierten nicht-kleinzelligen Lungenkarzinoms). https://www.g-ba.de/downloads/39-261-3646/2019-01-17_AM-RL-XII_Osimertinib_D-369_BAnz.pdf. Accessed 08 February 2022

[56]: Soria J, Ohe Y, Vansteenkiste J, Reungwetwattana T, Chewaskulyong B, Lee KH, Dechaphunkul A, Imamura F, Nogami N, Kurata T, Okamoto I, Zhou C, Cho BC, Cheng Y, Cho EK, Voon PJ, Planchard D, Su W, Gray JE, Lee S, Hodge R, Marotti M, Rukazenkov Y, Ramalingam SS (2018) Osimertinib in Untreated EGFR-Mutated Advanced Non-Small-Cell Lung Cancer. N Engl J Med *378,* 113-125. DOI: 10.1056/NEJMoa1713137

[57]: Institut für Qualität und Wirtschaftlichkeit im Gesundheitswesen (IQWiG) (2021) Osimertinib (NSCLC, adjuvant) - Nutzenbewertung gemäß § 35a SGB V. https://www.g-ba.de/downloads/92-975-4912/2021-07-01_Nutzenbewertung-IQWiG_Osimertinib_D-701.pdf. Accessed 08 February 2022

[58]: Gemeinsamer Bundesausschuss (GBA) (2021) Beschluss des Gemeinsamen Bundesausschusses über eine Änderung der Arzneimittel-Richtlinie (AM-RL): Anlage XII - Beschlüsse über die Nutzenbewertung von Arzneimitteln mit neuen Wirkstoffen nach § 35a SGB V - Osimertinib (neues Anwendungsgebiet: Nicht-kleinzelliges Lungenkarzinom, EGFR Mutation, adjuvante Therapie). https://www.g-ba.de/downloads/39-261-5177/2021-12-16_AM-RL-XII_Osimertinib_D-701.pdf. Accessed 08 February 2022

[59]: Wu Y, Tsuboi M, He J, John T, Grohe C, Majem M, Goldman JW, Laktionov K, Kim S, Kato T, Vu H, Lu S, Lee K, Akewanlop C, Yu C, de Marinis F, Bonanno L, Domine M, Shepherd FA, Zeng L, Hodge R, Atasoy A, Rukazenkov Y, Herbst RS (2020) Osimertinib in Resected EGFR-Mutated Non-Small-Cell Lung Cancer. N Engl J Med *383,* 1711-1723. DOI: 10.1056/NEJMoa2027071

[60]: Institut für Qualität und Wirtschaftlichkeit im Gesundheitswesen (IQWiG) (2017) Palbociclib (Mammakarzinom) - Nutzenbewertung gemäß § 35a SGB V. https://www.g-ba.de/downloads/92-975-1746/2017-03-01_Nutzenbewertung-IQWiG_Palbociclib-D-264.pdf. Accessed 08 February 2022

[61]: Gemeinsamer Bundesausschuss (GBA) (2017) Beschluss des Gemeinsamen Bundesausschusses über eine Änderung der Arzneimittel-Richtlinie (AM-RL): Anlage XII - Beschlüsse über die Nutzenbewertung von Arzneimitteln mit neuen Wirkstoffen nach § 35a SGB V - Palbociclib. https://www.g-ba.de/downloads/39-261-2947/2017-05-18_AM-RL-XII_Palbociclib_D-264_BAnz.pdf. Accessed 08 February 2022

[62]: Finn RS, Crown JP, Lang I, Boer K, Bondarenko IM, Kulyk SO, Ettl J, Patel R, Pinter T, Schmidt M, Shparyk Y, Thummala AR, Voytko NL, Fowst C, Huang X, Kim ST, Randolph S, Slamon DJ (2015) The cyclin-dependent kinase 4/6 inhibitor palbociclib in combination with letrozole versus letrozole alone as first-line treatment of oestrogen receptor-positive, HER2-negative, advanced breast cancer (PALOMA-1/TRIO-18): a randomised phase 2 study. Lancet Oncol *16,* 25-35. DOI: 10.1016/S1470-2045(14)71159-3

[63]: Finn RS, Martin M, Rugo HS, Jones S, Im S, Gelmon K, Harbeck N, Lipatov ON, Walshe JM, Moulder S, Gauthier E, Lu DR, Randolph S, Diéras V, Slamon DJ (2016) Palbociclib and Letrozole in Advanced Breast Cancer. N Engl J Med *375,* 1925-1936. DOI: 10.1056/NEJMoa1607303

[64]: Cristofanilli M, Turner NC, Bondarenko I, Ro J, Im S, Masuda N, Colleoni M, DeMichele A, Loi S, Verma S, Iwata H, Harbeck N, Zhang K, Theall KP, Jiang Y, Bartlett CH, Koehler M, Slamon D (2016) Fulvestrant plus palbociclib versus fulvestrant plus placebo for treatment of hormone-receptor-positive, HER2-negative metastatic breast cancer that progressed on previous endocrine therapy (PALOMA-3): final analysis of the multicentre, double-blind, phase 3 randomised controlled trial. Lancet Oncol *17,* 425-439. DOI: 10.1016/S1470-2045(15)00613-0

[65]: Institut für Qualität und Wirtschaftlichkeit im Gesundheitswesen (IQWiG) (2019) Palbociclib (Mammakarzinom) - Nutzenbewertung gemäß § 35a SGB V (Ablauf Befristung). https://www.g-ba.de/downloads/92-975-2610/2018-10-01_Nutzenbewertung-IQWiG_Palbociclib-D-395.pdf. Accessed 08 February 2022

[66]: Gemeinsamer Bundesausschuss (GBA) (2019) Beschluss des Gemeinsamen Bundesausschusses über eine Änderung der Arzneimittel-Richtlinie (AM-RL): Anlage XII - Beschlüsse über die Nutzenbewertung von Arzneimitteln mit neuen Wirkstoffen nach § 35a SGB V - Palbociclib (Brustkrebs; in Kombination mit Fulvestrant nach endokriner Therapie; Neubewertung nach Fristablauf). https://www.g-ba.de/downloads/39-261-3713/2019-03-22_AM-RL-XII_Palbociclib_D-395_BAnz.pdf. Accessed 08 February 2022

[67]: Institut für Qualität und Wirtschaftlichkeit im Gesundheitswesen (IQWiG) (2017) Alectinib (nicht kleinzelliges Lungenkarzinom) - Nutzenbewertung gemäß § 35a SGB V. https://www.g-ba.de/downloads/92-975-1918/2017-05-01_Nutzenbewertung-IQWiG_Alectinib_D-281.pdf. Accessed 08 February 2022

[68]: Institut für Qualität und Wirtschaftlichkeit im Gesundheitswesen (IQWiG) (2017) Alectinib (nicht kleinzelliges Lungenkarzinom) - Addendum zum Auftrag A17-19. https://www.g-ba.de/downloads/92-975-2000/2017-10-19_AM-RL-XII_Alectinib_D-281_Addendum.pdf. Accessed 08 February 2022

[69]: Gemeinsamer Bundesausschuss (GBA) (2017) Beschluss des Gemeinsamen Bundesausschusses über eine Änderung der Arzneimittel-Richtlinie (AM-RL): Anlage XII - Beschlüsse über die Nutzenbewertung von Arzneimitteln mit neuen Wirkstoffen nach § 35a SGB V - Alectinib. https://www.g-ba.de/downloads/39-261-3090/2017-10-19_AM-RL-XII_Alectinib_D-281_BAnz.pdf. Accessed 08 February 2022

[70]: Novello S, Mazières J, Oh I, de Castro J, Migliorino MR, Helland Å, Dziadziuszko R, Griesinger F, Kotb A, Zeaiter A, Cardona A, Balas B, Johannsdottir HK, Das-Gupta A, Wolf J (2018) Alectinib versus chemotherapy in crizotinib-pretreated anaplastic lymphoma kinase (ALK)-positive non-small-cell lung cancer: results from the phase III ALUR study. Ann Oncol *29,* 1409-1416. DOI: 10.1093/annonc/mdy121

[71]: Institut für Qualität und Wirtschaftlichkeit im Gesundheitswesen (IQWiG) (2018) Alectinib (nicht kleinzelliges Lungenkarzinom) - Nutzenbewertung gemäß § 35a SGB V (neues Anwendungsgebiet). https://www.g-ba.de/downloads/92-975-2247/2018-01-01_Nutzenbewertung-IQWiG_Alectinib-D-326.pdf. Accessed 08 February 2022

[72]: Gemeinsamer Bundesausschuss (GBA) (2018) Beschluss des Gemeinsamen Bundesausschusses über eine Änderung der Arzneimittel-Richtlinie (AM-RL): Anlage XII - Beschlüsse über die Nutzenbewertung von Arzneimitteln mit neuen Wirkstoffen nach § 35a SGB V - Alectinib (neues Anwendungsgebiet: Erstlinienbehandlung nicht-kleinzelliges Lungenkarzinom). https://www.g-ba.de/downloads/39-261-3368/2018-06-21_AM-RL-XII_Alectinib_D-326_BAnz.pdf. Accessed 08 February 2022

[73]: Peters S, Camidge DR, Shaw AT, Gadgeel S, Ahn JS, Kim D, Ou SI, Pérol M, Dziadziuszko R, Rosell R, Zeaiter A, Mitry E, Golding S, Balas B, Noe J, Morcos PN, Mok T (2017) Alectinib versus Crizotinib in Untreated ALK-Positive Non-Small-Cell Lung Cancer. N Engl J Med *377,* 829-838. DOI: 10.1056/NEJMoa1704795

[74]: Gemeinsamer Bundesausschuss (GBA) (2018) Nutzenbewertung von Arzneimittel mit neuen Wirkstoffen nach § 35a SGB V, Bewertung von Arzneimitteln für seltene Leiden nach § 35a Absatz 1 Satz 10 i.V.m. 5. Kapitel § 12 Nr. 1 Satz 2 VerfO, Wirkstoff: Midostaurin (Teil A). https://www.g-ba.de/downloads/92-975-2154/2017-10-15_Nutzenbewertung-G-BA-Teil-A_Midostaurin-D-319.pdf. Accessed 08 February 2022

[75]: Gemeinsamer Bundesausschuss (GBA) (2018) Beschluss des Gemeinsamen Bundesausschusses über eine Änderung der Arzneimittel-Richtlinie (AM-RL): Anlage XII - Beschlüsse über die Nutzenbewertung von Arzneimitteln mit neuen Wirkstoffen nach § 35a SGB V - Midostaurin (akute myeloische Leukämie). https://www.g-ba.de/downloads/39-261-3277/2018-04-05_AM-RL-XII_Midostaurin_AML_D-319_BAnz.pdf. Accessed 08 February 2022

[76]: Stone RM, Mandrekar SJ, Sanford BL, Laumann K, Geyer S, Bloomfield CD, Thiede C, Prior TW, Döhner K, Marcucci G, Lo-Coco F, Klisovic RB, Wei A, Sierra J, Sanz MA, Brandwein JM, de Witte T, Niederwieser D, Appelbaum FR, Medeiros BC, Tallman MS, Krauter J, Schlenk RF, Ganser A, Serve H, et al (2017) Midostaurin plus Chemotherapy for Acute Myeloid Leukemia with a FLT3 Mutation. N Engl J Med *377,* 454-464. DOI: 10.1056/NEJMoa1614359

[77]: Gemeinsamer Bundesausschuss (GBA) (2018) Nutzenbewertung von Arzneimittel mit neuen Wirkstoffen nach § 35a SGB V, Bewertung von Arzneimitteln für seltene Leiden nach § 35a Absatz 1 Satz 10 i.V.m. 5. Kapitel § 12 Nr. 1 Satz 2 VerfO, Wirkstoff: Midostaurin (AWG B). https://www.g-ba.de/downloads/92-975-2156/2017-10-15_Nutzenbewertung-G-BA-Teil-B_Midostaurin-D-319.pdf. Accessed 08 February 2022

[78]: Gemeinsamer Bundesausschuss (GBA) (2018) Beschluss des Gemeinsamen Bundesausschusses über eine Änderung der Arzneimittel-Richtlinie (AM-RL): Anlage XII - Beschlüsse über die Nutzenbewertung von Arzneimitteln mit neuen Wirkstoffen nach § 35a SGB V - Midostaurin (aggressive systemische Mastozytose). https://www.g-ba.de/downloads/39-261-3279/2018-04-05_AM-RL-XII_Midostaurin_ASM_D-319_BAnz.pdf. Accessed 08 February 2022

[79]: Gotlib J, Kluin-Nelemans HC, George TI, Akin C, Sotlar K, Hermine O, Awan FT, Hexner E, Mauro MJ, Sternberg DW, Villeneuve M, Huntsman Labed A, Stanek EJ, Hartmann K, Horny H, Valent P, Reiter A (2016) Efficacy and Safety of Midostaurin in Advanced Systemic Mastocytosis. N Engl J Med *374,* 2530-2541. DOI: 10.1056/NEJMoa1513098

[80]: DeAngelo DJ, George TI, Linder A, Langford C, Perkins C, Ma J, Westervelt P, Merker JD, Berube C, Coutre S, Liedtke M, Medeiros B, Sternberg D, Dutreix C, Ruffie P, Corless C, Graubert TJ, Gotlib J (2018). Efficacy and safety of midostaurin in patients with advanced systemic mastocytosis: 10-year median follow-up of a phase II trial. Leukemia *32,* 470-478. DOI: 10.1038/leu.2017.234

[81]: Institut für Qualität und Wirtschaftlichkeit im Gesundheitswesen (IQWiG) (2017) Ribociclib (Mammakarzinom) - Nutzenbewertung gemäß § 35a SGB V. https://www.g-ba.de/downloads/92-975-2079/2017-12-15_Nutzenbewertung-IQWiG_Ribociclib-D-307.pdf. Accessed 08 February 2022

[82]: Gemeinsamer Bundesausschuss (GBA) (2018) Beschluss des Gemeinsamen Bundesausschusses über eine Änderung der Arzneimittel-Richtlinie (AM-RL): Anlage XII - Beschlüsse über die Nutzenbewertung von Arzneimitteln mit neuen Wirkstoffen nach § 35a SGB V - Ribociclib. https://www.g-ba.de/downloads/39-261-3253/2018-03-16_AM-RL-XII_Ribociclib_D-307_BAnz.pdf. Accessed 08 February 2022

[83]: Hortobagyi GN, Stemmer SM, Burris HA, Yap Y, Sonke GS, Paluch-Shimon S, Campone M, Blackwell KL, André F, Winer EP, Janni W, Verma S, Conte P, Arteaga CL, Cameron DA, Petrakova K, Hart LL, Villanueva C, Chan A, Jakobsen E, Nusch A, Burdaeva O, Grischke E, Alba E, Wist E, et al (2016) Ribociclib as First-Line Therapy for HR-Positive, Advanced Breast Cancer. N Engl J Med *375,* 1738-1748. DOI: 10.1056/NEJMoa1609709

[84]: Institut für Qualität und Wirtschaftlichkeit im Gesundheitswesen (IQWiG) (2020) Ribociclib (Mammakarzinom, Kombination mit einem Aromatasehemmer) - Nutzenbewertung gemäß § 35a SGB V (Ablauf Befristung). https://www.g-ba.de/downloads/92-975-3555/2020-03-01_Nutzenbewertung-IQWiG_Ribociclib_D-517.pdf. Accessed 09 February 2022

[85]: Gemeinsamer Bundesausschuss (GBA) (2020) Beschluss des Gemeinsamen Bundesausschusses über eine Änderung der Arzneimittel-Richtlinie (AM-RL): Anlage XII - Nutzenbewertung von Arzneimitteln mit neuen Wirkstoffen nach § 35a SGB V - Ribociclib (Neubewertung nach Fristablauf (Mammakarzinom, HR+, HER2-, Kombination mit einem Aromatasehemmer)). https://www.g-ba.de/downloads/39-261-4423/2020-08-20_AM- RL-XII_Ribociclib_D-517_BAnz.pdf. Accessed 09 February 2022

[86]: Hortobagyi GN, Stemmer SM, Burris HA, Yap YS, Sonke GS, Paluch-Shimon S, Campone M, Petrakova K, Blackwell KL, Winer EP, Janni W, Verma S, Conte P, Arteaga CL, Cameron DA, Mondal S, Su F, Miller M, Elmeliegy M, Germa C, O'Shaughnessy J (2018) Updated results from MONALEESA-2, a phase III trial of first-line ribociclib plus letrozole versus placebo plus letrozole in hormone receptor-positive, HER2-negative advanced breast cancer. Ann Oncol *29,* 1541-1547. DOI: 10.1093/annonc/mdy155

[87]: Institut für Qualität und Wirtschaftlichkeit im Gesundheitswesen (IQWiG) (2019) Ribociclib (Mammakarzinom) - Nutzenbewertung gemäß § 35a SGB V. https://www.g-ba.de/downloads/92-975-2867/2019-01-15_Nutzenbewertung-IQWiG_Ribociclib-D-430.pdf. Accessed 09 February 2022

[88]: Gemeinsamer Bundesausschuss (GBA) (2019) Beschluss des Gemeinsamen Bundesausschusses über eine Änderung der Arzneimittel-Richtlinie (AM-RL): Anlage XII - Beschlüsse über die Nutzenbewertung von Arzneimitteln mit neuen Wirkstoffen nach § 35a SGB V - Ribociclib (neues Anwendungsgebiet: Brustkrebs, in Kombination mit Fulvestrant). https://www.g-ba.de/downloads/39-261-3863/2019-07-04_AM-RL-XII_Ribociclib-Fulvestrant_D-430_BAnz.pdf. Accessed 09 February 2022

[89]: Slamon DJ, Neven P, Chia S, Fasching PA, De Laurentiis M, Im S, Petrakova K, Bianchi GV, Esteva FJ, Martín M, Nusch A, Sonke GS, De la Cruz-Merino L, Beck JT, Pivot X, Vidam G, Wang Y, Rodriguez Lorenc K, Miller M, Taran T, Jerusalem G (2018) Phase III Randomized Study of Ribociclib and Fulvestrant in Hormone Receptor-Positive, Human Epidermal Growth Factor Receptor 2-Negative Advanced Breast Cancer: MONALEESA-3. J Clin Oncol *36,* 2465-2472. DOI: 10.1200/JCO.2018.78.9909

[90]: Institut für Qualität und Wirtschaftlichkeit im Gesundheitswesen (IQWiG) (2020) Ribociclib (Mammakarzinom, Kombination mit Fulvestrant) - Nutzenbewertung gemäß § 35a SGB V (Ablauf Befristung). https://www.g-ba.de/downloads/92-975-3567/2020-03-01_Nutzenbewertung-IQWiG_Ribociclib_D-518.pdf. Accessed 09 February 2022

[91]: Gemeinsamer Bundesausschuss (GBA) (2020) Beschluss des Gemeinsamen Bundesausschusses über eine Änderung der Arzneimittel-Richtlinie (AM-RL): Anlage XII - Nutzenbewertung von Arzneimitteln mit neuen Wirkstoffen nach § 35a SGB V - Ribociclib (Neubewertung nach Fristablauf (Mammakarzinom, HR+, HER2-, Kombination mit Fulvestrant)). https://www.g-ba.de/downloads/39-261-4428/2020-08-20_AM-RL-XII_Ribociclib_D-518_BAnz.pdf. Accessed 09 February 2022

[92]: Slamon DJ, Neven P, Chia S, Fasching PA, De Laurentiis M, Im S, Petrakova K, Bianchi GV, Esteva FJ, Martín M, Nusch A, Sonke GS, De la Cruz-Merino L, Beck JT, Pivot X, Sondhi M, Wang Y, Chakravartty A, Rodriguez-Lorenc K, Taran T, Jerusalem G (2020) Overall Survival with Ribociclib plus Fulvestrant in Advanced Breast Cancer. N Engl J Med *382,* 514-524. DOI: 10.1056/NEJMoa1911149

[93]: Institut für Qualität und Wirtschaftlichkeit im Gesundheitswesen (IQWiG) (2018) Tivozanib (Nierenzellkarzinom) - Nutzenbewertung gemäß § 35a SGB V. https://www.g-ba.de/downloads/92-975-2163/2017-11-01_Nutzenbewertung-IQWiG_Tivozanib_D-323.pdf. Accessed 15 February 2022

[94]: Gemeinsamer Bundesausschuss (GBA) (2018) Beschluss des Gemeinsamen Bundesausschusses über eine Änderung der Arzneimittel-Richtlinie (AM-RL): Anlage XII - Beschlüsse über die Nutzenbewertung von Arzneimitteln mit neuen Wirkstoffen nach § 35a SGB V - Tivozanib. https://www.g-ba.de/downloads/39-261-3291/2018-04-19_AM-RL-XII_Tivozanib_D-323_BAnz.pdf. Accessed 15 February 2022

[95]: Motzer RJ, Nosov D, Eisen T, Bondarenko I, Lesovoy V, Lipatov O, Tomczak P, Lyulko O, Alyasova A, Harza M, Kogan M, Alekseev BY, Sternberg CN, Szczylik C, Cella D, Ivanescu C, Krivoshik A, Strahs A, Esteves B, Berkenblit A, Hutson TE (2013) Tivozanib versus sorafenib as initial targeted therapy for patients with metastatic renal cell carcinoma: results from a phase III trial. J Clin Oncol *31,* 3791-3799. DOI: 10.1200/JCO.2012.47.4940

[96]: Eichelberg C, Vervenne WL, De Santis M, Fischer von Weikersthal L, Goebell PJ, Lerchenmüller C, Zimmermann U, Bos MMEM, Freier W, Schirrmacher-Memmel S, Staehler M, Pahernik S, Los M, Schenck M, Flörcken A, van Arkel C, Hauswald K, Indorf M, Gottstein D, Michel MS (2015) SWITCH: A Randomised, Sequential, Open-label Study to Evaluate the Efficacy and Safety of Sorafenib-sunitinib Versus Sunitinib-sorafenib in the Treatment of Metastatic Renal Cell Cancer. Eur Urol *68,* 837-847. DOI: 10.1016/j.eururo.2015.04.017

[97]: Haas NB, Manola J, Uzzo RG, Flaherty KT, Wood CG, Kane C, Jewett M, Dutcher JP, Atkins MB, Pins M, Wilding G, Cella D, Wagner L, Matin S, Kuzel TM, Sexton WJ, Wong Y, Choueiri TK, Pili R, Puzanov I, Kohli M, Stadler W, Carducci M, Coomes R, DiPaola RS (2016) Adjuvant sunitinib or sorafenib for high-risk, non-metastatic renal-cell carcinoma (ECOG-ACRIN E2805): a double-blind, placebo-controlled, randomised, phase 3 trial. Lancet *387,* 2008-2016. DOI: 10.1016/S0140-6736(16)00559-6

[98]: Institut für Qualität und Wirtschaftlichkeit im Gesundheitswesen (IQWiG) (2019) Abemaciclib (Mammakarzinom; Kombination mit einem Aromatasehemmer) - Nutzenbewertung gemäß § 35a SGB V. https://www.g-ba.de/downloads/92-975-2702/2018-11-01_Nutzenbewertung-IQWiG_Abemaciclib-D-400.pdf. Accessed 15 February 2022

[99]: Gemeinsamer Bundesausschuss (GBA) (2019) Beschluss des Gemeinsamen Bundesausschusses über eine Änderung der Arzneimittel-Richtlinie (AM-RL): Anlage XII - Beschlüsse über die Nutzenbewertung von Arzneimitteln mit neuen Wirkstoffen nach § 35a SGB V - Abemaciclib (Brustkrebs; in Kombination mit einem Aromatasehemmer). https://www.g-ba.de/downloads/39-261-3767/2019-05-02_AM-RL-XII_Abemaciclib_D-400_BAnz.pdf. Accessed 15 February 2022

[100]: Goetz MP, Toi M, Campone M, Sohn J, Paluch-Shimon S, Huober J, Park IH, Trédan O, Chen S, Manso L, Freedman OC, Garnica Jaliffe G, Forrester T, Frenzel M, Barriga S, Smith IC, Bourayou N, Di Leo A (2017) MONARCH 3: Abemaciclib As Initial Therapy for Advanced Breast Cancer. J Clin Oncol *35,* 3638-3646. DOI: 10.1200/JCO.2017.75.6155

[101]: Institut für Qualität und Wirtschaftlichkeit im Gesundheitswesen (IQWiG) (2019) Abemaciclib (Mammakarzinom; Kombination mit Fulvestrant) - Nutzenbewertung gemäß § 35a SGB V. https://www.g-ba.de/downloads/92-975-2708/2018-11-01_Nutzenbewertung-IQWiG_Abemaciclib-D-401.pdf. Accessed 15 February 2022

[102]: Gemeinsamer Bundesausschuss (GBA) (2019) Beschluss des Gemeinsamen Bundesausschusses über eine Änderung der Arzneimittel-Richtlinie (AM-RL): Anlage XII - Beschlüsse über die Nutzenbewertung von Arzneimitteln mit neuen Wirkstoffen nach § 35a SGB V - Abemaciclib (Brustkrebs; in Kombination mit Fulvestrant). https://www.g-ba.de/downloads/39-261-3768/2019-05-02_AM-RL-XII_Abemaciclib_D-401_BAnz.pdf. Accessed 15 February 2022

[103]: Sledge GW, Toi M, Neven P, Sohn J, Inoue K, Pivot X, Burdaeva O, Okera M, Masuda N, Kaufman PA, Koh H, Grischke E, Frenzel M, Lin Y, Barriga S, Smith IC, Bourayou N, Llombart-Cussac A (2017) MONARCH 2: Abemaciclib in Combination With Fulvestrant in Women With HR+/HER2- Advanced Breast Cancer Who Had Progressed While Receiving Endocrine Therapy. J Clin Oncol *35,* 2875-2884. DOI: 10.1200/JCO.2017.73.7585

[104]: Institut für Qualität und Wirtschaftlichkeit im Gesundheitswesen (IQWiG) (2020) Abemaciclib (Mammakarzinom; Kombination mit Fulvestrant) - Nutzenbewertung gemäß § 35a SGB V (Ablauf Befristung). https://www.g-ba.de/downloads/92-975-3609/2020-03-15_Nutzenbewertung-IQWiG_Abemaciclib_D-531.pdf. Accessed 15 February 2022

[105]: Gemeinsamer Bundesausschuss (GBA) (2020) Beschluss des Gemeinsamen Bundesausschusses über eine Änderung der Arzneimittel-Richtlinie (AM-RL): Anlage XII - Nutzenbewertung von Arzneimitteln mit neuen Wirkstoffen nach § 35a SGB V - Abemaciclib (Neubewertung nach Fristablauf: Mammakarzinom, HR+, HER2-, Kombination mit Fulvestrant). https://www.g-ba.de/downloads/39-261-4443/2020-09-03_AM-RL-XII_Abemaciclib_D-531_BAnz.pdf. Accessed 15 February 2022

[106]: Sledge GW, Toi M, Neven P, Sohn J, Inoue K, Pivot X, Burdaeva O, Okera M, Masuda N, Kaufman PA, Koh H, Grischke E, Conte P, Lu Y, Barriga S, Hurt K, Frenzel M, Johnston S, Llombart-Cussac A (2020) The Effect of Abemaciclib Plus Fulvestrant on Overall Survival in Hormone Receptor-Positive, ERBB2-Negative Breast Cancer That Progressed on Endocrine Therapy-MONARCH 2: A Randomized Clinical Trial. JAMA Oncol *6,* 116-124. DOI: 10.1001/jamaoncol.2019.4782

[107|: Zhang QY, Sun T, Yin YM, Li HP, Yan M, Tong ZS, Oppermann CP, Liu YP, Costa R, Li M, Cheng Y, Ouyang QC, Chen X, Liao N, Wu XH, Wang XJ, Feng JF, Hegg R, Kanakasetty GB, Coccia-Portugal MA, Han RB, Lu Y, Chi HD, Jiang ZF, Hu XC (2020) MONARCH plus: abemaciclib plus endocrine therapy in women with HR+/HER2- advanced breast cancer: the multinational randomized phase III study. Ther Adv Med Oncol *12,* 1758835920963925. DOI: 10.1177/1758835920963925

[108]: Institut für Qualität und Wirtschaftlichkeit im Gesundheitswesen (IQWiG) (2019) Binimetinib (Melanom) - Nutzenbewertung gemäß § 35a SGB V. https://www.g-ba.de/downloads/92-975-2618/2018-10-01_Nutzenbewertung-IQWiG_Binimetinib-D-388.pdf. Accessed 15 February 2022

[109]: Gemeinsamer Bundesausschuss (GBA) (2019) Beschluss des Gemeinsamen Bundesausschusses über eine Änderung der Arzneimittel-Richtlinie (AM-RL): Anlage XII - Beschlüsse über die Nutzenbewertung von Arzneimitteln mit neuen Wirkstoffen nach § 35a SGB V - Binimetinib. https://www.g-ba.de/downloads/39-261-3725/2019-03-22_AM-RL-XII_Binimetinib_D-388_BAnz.pdf. Accessed 15 February 2022

[110]: Dummer R, Ascierto PA, Gogas HJ, Arance A, Mandala M, Liszkay G, Garbe C, Schadendorf D, Krajsova I, Gutzmer R, Chiarion-Sileni V, Dutriaux C, de Groot JWB, Yamazaki N, Loquai C, Moutouh-de Parseval LA, Pickard MD, Sandor V, Robert C, Flaherty KT (2018) Encorafenib plus binimetinib versus vemurafenib or encorafenib in patients with BRAF-mutant melanoma (COLUMBUS): a multicentre, open-label, randomised phase 3 trial. Lancet Oncol *19,* 603-615. DOI: 10.1016/S1470-2045(18)30142-6

[111]: Institut für Qualität und Wirtschaftlichkeit im Gesundheitswesen (IQWiG) (2019) Encorafenib (Melanom) - Nutzenbewertung gemäß § 35a SGB V. https://www.g-ba.de/downloads/92-975-2611/2018-10-01_Nutzenbewertung-IQWiG_Encorafenib-D-389.pdf. Accessed 15 February 2022

[112]: Gemeinsamer Bundesausschuss (GBA) (2019) Beschluss des Gemeinsamen Bundesausschusses über eine Änderung der Arzneimittel-Richtlinie (AM-RL): Anlage XII - Beschlüsse über die Nutzenbewertung von Arzneimitteln mit neuen Wirkstoffen nach § 35a SGB V - Encorafenib. https://www.g-ba.de/downloads/39-261-3710/2019-03-22_AM-RL-XII_Encorafenib_D-389_BAnz.pdf. Accessed 15 February 2022

[113]: Institut für Qualität und Wirtschaftlichkeit im Gesundheitswesen (IQWiG) (2020) Encorafenib (Kolorektalkarzinom) - Nutzenbewertung gemäß § 35a SGB V. https://www.g-ba.de/downloads/92-975-3823/2020-07-01_Nutzenbewertung-IQWiG_Encorafenib-D-551.pdf. Accessed 15 February 2022

[114]: Gemeinsamer Bundesausschuss (GBA) (2020) Beschluss des Gemeinsamen Bundesausschusses über eine Änderung der Arzneimittel-Richtlinie (AM-RL): Anlage XII - Nutzenbewertung von Arzneimitteln mit neuen Wirkstoffen nach § 35a SGB V - Encorafenib (neues Anwendungsgebiet: metastasiertes Kolorektalkarzinom mit BRAF-V600E-Mutation nach systemischer Vortherapie; in Kombination mit Cetuximab). https://www.g-ba.de/downloads/39-261-4609/2020-12-17_AM-RL-XII_Encorafenib_D-551_BAnz.pdf. Accessed 15 February 2022

[115]: Kopetz S, Grothey A, Yaeger R, Van Cutsem E, Desai J, Yoshino T, Wasan H, Ciardiello F, Loupakis F, Hong YS, Steeghs N, Guren TK, Arkenau H, Garcia-Alfonso P, Pfeiffer P, Orlov S, Lonardi S, Elez E, Kim T, Schellens JHM, Guo C, Krishnan A, Dekervel J, Morris V, Calvo Ferrandiz A, et al (2019) Encorafenib, Binimetinib, and Cetuximab in BRAF V600E-Mutated Colorectal Cancer. N Engl J Med *381,* 1632-1643. DOI: 10.1056/NEJMoa1908075

[116]: Institut für Qualität und Wirtschaftlichkeit im Gesundheitswesen (IQWiG) (2019) Brigatinib (NSCLC) - Nutzenbewertung gemäß § 35a SGB V. https://www.g-ba.de/downloads/92-975-2883/2019-01-15_Nutzenbewertung-IQWiG_Brigatinib-D-434.pdf. Accessed 15 February 2022

[117]: Gemeinsamer Bundesausschuss (GBA) (2019) Beschluss des Gemeinsamen Bundesausschusses über eine Änderung der Arzneimittel-Richtlinie (AM-RL): Anlage XII - Beschlüsse über die Nutzenbewertung von Arzneimitteln mit neuen Wirkstoffen nach § 35a SGB V - Brigatinib. https://www.g-ba.de/downloads/39-261-3859/2019-07-04_AM-RL-XII_Brigatinib_D-434_BAnz.pdf. Accessed 15 February 2022

[118]: Kim D, Tiseo M, Ahn M, Reckamp KL, Hansen KH, Kim S, Huber RM, West HL, Groen HJM, Hochmair MJ, Leighl NB, Gettinger SN, Langer CJ, Paz-Ares Rodríguez LG, Smit EF, Kim ES, Reichmann W, Haluska FG, Kerstein D, Camidge DR (2017) Brigatinib in Patients With Crizotinib-Refractory Anaplastic Lymphoma Kinase-Positive Non-Small-Cell Lung Cancer: A Randomized, Multicenter Phase II Trial. J Clin Oncol *35,* 2490-2498. DOI: 10.1200/JCO.2016.71.5904

[119]: Gettinger SN, Bazhenova LA, Langer CJ, Salgia R, Gold KA, Rosell R, Shaw AT, Weiss GJ, Tugnait M, Narasimhan NI, Dorer DJ, Kerstein D, Rivera VM, Clackson T, Haluska FG, Camidge DR (2016) Activity and safety of brigatinib in ALK-rearranged non-small-cell lung cancer and other malignancies: a single-arm, open-label, phase 1/2 trial. Lancet Oncol *17,* 1683-1696. DOI: 10.1016/S1470-2045(16)30392-8

[120]: Camidge DR, Kim HR, Ahn M, Yang JC, Han J, Lee J, Hochmair MJ, Li JY, Chang G, Lee KH, Gridelli C, Delmonte A, Garcia Campelo R, Kim D, Bearz A, Griesinger F, Morabito A, Felip E, Califano R, Ghosh S, Spira A, Gettinger SN, Tiseo M, Gupta N, Haney J, et al (2018) Brigatinib versus Crizotinib in ALK-Positive Non-Small-Cell Lung Cancer. N Engl J Med *379,* 2027-2039. DOI: 10.1056/NEJMoa1810171

[121]: Institut für Qualität und Wirtschaftlichkeit im Gesundheitswesen (IQWiG) (2020) Brigatinib (nicht kleinzelliges Lungenkarzinom) - Nutzenbewertung gemäß § 35a SGB V. https://www.g-ba.de/downloads/92-975-3700/2020-05-01_Nutzenbewertung-IQWiG_Brigatinib_D-542.pdf. Accessed 15 February 2022

[122]: Gemeinsamer Bundesausschuss (GBA) (2020) Beschluss des Gemeinsamen Bundesausschusses über eine Änderung der Arzneimittel-Richtlinie (AM-RL): Anlage XII - Nutzenbewertung von Arzneimitteln mit neuen Wirkstoffen nach § 35a SGB V - Brigatinib (neues Anwendungsgebiet: NSCLC, ALK+, ALK-Inhibitor-naive Patienten). https://www.g-ba.de/downloads/39-261-4498/2020-10-15_AM-RL_XII_Brigatinib_D-542_BAnz.pdf. Accessed 15 February 2022

[123]: Institut für Qualität und Wirtschaftlichkeit im Gesundheitswesen (IQWiG) (2019) Dacomitinib (nicht kleinzelliges Lungenkarzinom) - Nutzenbewertung gemäß § 35a SGB V. https://www.g-ba.de/downloads/92-975-3070/2019-05-01_Nutzenbewertung-IQWiG_Dacomitinib-D-442.pdf. Accessed 19 February 2022

[124]: Gemeinsamer Bundesausschuss (GBA) (2019) Beschluss des Gemeinsamen Bundesausschusses über eine Änderung der Arzneimittel-Richtlinie (AM-RL): Anlage XII - Nutzenbewertung von Arzneimitteln mit neuen Wirkstoffen nach § 35a SGB V - Dacomitinib. https://www.g-ba.de/downloads/39-261-3992/2019-10-17_AM-RL-XII_Dacomitinib_D-442_BAnz.pdf. Accessed 19 February 2022

[125]: Wu Y, Cheng Y, Zhou X, Lee KH, Nakagawa K, Niho S, Tsuji F, Linke R, Rosell R, Corral J, Migliorino MR, Pluzanski A, Sbar EI, Wang T, White JL, Nadanaciva S, Sandin R, Mok TS (2017) Dacomitinib versus gefitinib as first-line treatment for patients with EGFR-mutation-positive non-small-cell lung cancer (ARCHER 1050): a randomised, open-label, phase 3 trial. Lancet Oncol *18,* 1454-1466. DOI: 10.1016/S1470-2045(17)30608-3

[126]: Mok TS, Cheng Y, Zhou X, Lee KH, Nakagawa K, Niho S, Lee M, Linke R, Rosell R, Corral J, Migliorino MR, Pluzanski A, Sbar EI, Wang T, White JL, Wu Y (2018) Improvement in Overall Survival in a Randomized Study That Compared Dacomitinib With Gefitinib in Patients With Advanced Non-Small-Cell Lung Cancer and EGFR-Activating Mutations. J Clin Oncol *36,* 2244-2250. DOI: 10.1200/JCO.2018.78.7994

[127]: Gemeinsamer Bundesausschuss (GBA) (2020) Nutzenbewertung von Arzneimittel mit neuen Wirkstoffen nach § 35a SGB V, Bewertung von Arzneimitteln für seltene Leiden nach § 35a Absatz 1 Satz 11 i.V.m. 5. Kapitel § 12 Nr. 1 Satz 2 VerfO, Wirkstoff: Gilteritinib. https://www.g-ba.de/downloads/92-975-3452/2019-12-01_Nutzenbewertung-G-BA_Gilteritinib_D-503.pdf. Accessed 19 February 2022

[128]: Gemeinsamer Bundesausschuss (GBA) (2020) Beschluss des Gemeinsamen Bundesausschusses über eine Änderung der Arzneimittel-Richtlinie (AM-RL): Anlage XII - Nutzenbewertung von Arzneimitteln mit neuen Wirkstoffen nach § 35a SGB V - Gilteritinib (rezidivierte oder refraktäre akute myeloische Leukämie mit FLT3-Mutation). https://www.g-ba.de/downloads/39-261-4287/2020-05-14_AM-RL-XII_Gilteritinib_D-503_BAnz.pdf. Accessed 19 February 2022

[129]: Perl AE, Martinelli G, Cortes JE, Neubauer A, Berman E, Paolini S, Montesinos P, Baer MR, Larson RA, Ustun C, Fabbiano F, Erba HP, Di Stasi A, Stuart R, Olin R, Kasner M, Ciceri F, Chou W, Podoltsev N, Recher C, Yokoyama H, Hosono N, Yoon S, Lee J, Pardee T, et al (2019) Gilteritinib or Chemotherapy for Relapsed or Refractory FLT3-Mutated AML. N Engl J Med *381,* 1728-1740. DOI: 10.1056/NEJMoa1902688

[130]: Perl AE, Altman JK, Cortes J, Smith C, Litzow M, Baer MR, Claxton D, Erba HP, Gill S, Goldberg S, Jurcic JG, Larson RA, Liu C, Ritchie E, Schiller G, Spira AI, Strickland SA, Tibes R, Ustun C, Wang ES, Stuart R, Röllig C, Neubauer A, Martinelli G, Bahceci E, et al (2017) Selective inhibition of FLT3 by gilteritinib in relapsed or refractory acute myeloid leukaemia: a multicentre, first-in-human, open-label, phase 1-2 study. Lancet Oncol *18,* 1061-1075. DOI: 10.1016/S1470-2045(17)30416-3

[131]: Institut für Qualität und Wirtschaftlichkeit im Gesundheitswesen (IQWiG) (2020) Larotrectinib (solide Tumore mit einer neurotrophen Tyrosin-Rezeptor-Kinase [NTRK]- Genfusion) - Nutzenbewertung gemäß § 35a SGB V. https://www.g-ba.de/downloads/92-975-3355/2019-10-15_Nutzenbewertung-IQWiG_Larotrectinib_D-495.pdf. Accessed 19 February 2022

[132]: Gemeinsamer Bundesausschuss (GBA) (2020) Beschluss des Gemeinsamen Bundesausschusses über eine Änderung der Arzneimittel-Richtlinie (AM-RL): Anlage XII - Nutzenbewertung von Arzneimitteln mit neuen Wirkstoffen nach § 35a SGB V - Larotrectinib (solide Tumore, Histologie-unabhängig). https://www.g-ba.de/downloads/39-261-4242/2020-04-02_AM-RL-XII_Larotrectinib_D-495_BAnz.pdf. Accessed 19 February 2022

[133]: Hong DS, Bauer TM, Lee JJ, Dowlati A, Brose MS, Farago AF, Taylor M, Shaw AT, Montez S, Meric-Bernstam F, Smith S, Tuch BB, Ebata K, Cruickshank S, Cox MC, Burris HA, Doebele RC (2019) Larotrectinib in adult patients with solid tumours: a multi-centre, open-label, phase I dose-escalation study. Ann Oncol *30,* 325-331. DOI: 10.1093/annonc/mdy539

[134]: Drilon A, Tan DSW, Lassen UN, Leyvraz S, Liu Y, Patel JD, Rosen L, Solomon B, Norenberg R, Dima L, Brega N, Shen L, Moreno V, Kummar S, Lin JJ (2022) Efficacy and Safety of Larotrectinib in Patients With Tropomyosin Receptor Kinase Fusion-Positive Lung Cancers. JCO Precis Oncol *6,* e2100418. DOI: 10.1200/PO.21.00418

[135]: Laetsch TW, DuBois SG, Mascarenhas L, Turpin B, Federman N, Albert CM, Nagasubramanian R, Davis JL, Rudzinski E, Feraco AM, Tuch BB, Ebata KT, Reynolds M, Smith S, Cruickshank S, Cox MC, Pappo AS, Hawkins DS (2018) Larotrectinib for paediatric solid tumours harbouring NTRK gene fusions: phase 1 results from a multicentre, open-label, phase 1/2 study. Lancet Oncol *19,* 705-714. DOI: 10.1016/S1470-2045(18)30119-0

[136]: Institut für Qualität und Wirtschaftlichkeit im Gesundheitswesen (IQWiG) (2019) Lorlatinib (NSCLC) - Nutzenbewertung gemäß § 35a SGB V. https://www.g-ba.de/downloads/92-975-3096/2019-06-01_Nutzenbewertung-IQWiG_Lorlatinib_D-451.pdf. Accessed 19 February 2022

[137]: Gemeinsamer Bundesausschuss (GBA) (2019) Beschluss des Gemeinsamen Bundesausschusses über eine Änderung der Arzneimittel-Richtlinie (AM-RL): Anlage XII - Nutzenbewertung von Arzneimitteln mit neuen Wirkstoffen nach § 35a SGB V - Lorlatinib. https://www.g-ba.de/downloads/39-261-4029/2019-11-22_AM-RL-XII_Lorlatinib_D-451_BAnz.pdf. Accessed 19 February 2022

[138]: Solomon BJ, Besse B, Bauer TM, Felip E, Soo RA, Camidge DR, Chiari R, Bearz A, Lin C, Gadgeel SM, Riely GJ, Tan EH, Seto T, James LP, Clancy JS, Abbattista A, Martini J, Chen J, Peltz G, Thurm H, Ou SI, Shaw AT (2018) Lorlatinib in patients with ALK-positive non-small-cell lung cancer: results from a global phase 2 study. Lancet Oncol *19,* 1654-1667. DOI: 10.1016/S1470-2045(18)30649-1

[139]: Institut für Qualität und Wirtschaftlichkeit im Gesundheitswesen (IQWiG) (2020) Neratinib (Mammakarzinom) - Nutzenbewertung gemäß § 35a SGB V. https://www.g-ba.de/downloads/92-975-3446/2019-12-01_Nutzenbewertung-IQWiG_Neratinib_S-506.pdf. Accessed 19 February 2022

[140]: Gemeinsamer Bundesausschuss (GBA) (2020) Beschluss des Gemeinsamen Bundesausschusses über eine Änderung der Arzneimittel-Richtlinie (AM-RL): Anlage XII - Nutzenbewertung von Arzneimitteln mit neuen Wirkstoffen nach § 35a SGB V - Neratinib (Mammakarzinom, HR-positiv, HER2-positiv, adjuvante Behandlung). https://www.g-ba.de/downloads/39-261-4290/2020-05-14_AM-RL_XII_Neratinib_D-506_BAnz.pdf. Accessed 19 February 2022

[141]: Chan A, Delaloge S, Holmes FA, Moy B, Iwata H, Harvey VJ, Robert NJ, Silovski T, Gokmen E, von Minckwitz G, Ejlertsen B, Chia SKL, Mansi J, Barrios CH, Gnant M, Buyse M, Gore I, Smith J, Harker G, Masuda N, Petrakova K, Zotano AG, Iannotti N, Rodriguez G, Tassone P, et al (2016) Neratinib after trastuzumab-based adjuvant therapy in patients with HER2-positive breast cancer (ExteNET): a multicentre, randomised, double-blind, placebo-controlled, phase 3 trial. Lancet Oncol *17,* 367-377. DOI: 10.1016/S1470-2045(15)00551-3

[142]: Tian W, Zhang P, Yuan Y, Deng X, Yue R, Ge X (2020) Efficacy and safety of ceritinib in anaplastic lymphoma kinase-rearranged non-small cell lung cancer: A systematic review and meta-analysis. J Clin Pharm Ther *45,* 743-754. DOI: 10.1111/jcpt.13157

[143]: Cadranel J, Cortot AB, Lena H, Mennecier B, Do P, Dansin E, Mazieres J, Chouaid C, Perol M, Barlesi F, Robinet G, Friard S, Thiberville L, Audigier-Valette C, Vergnenegre A, Westeel V, Slimane K, Buturuga A, Moro-Sibilot D, Besse B (2018) Real-life experience of ceritinib in crizotinib-pretreated ALK+ advanced non-small cell lung cancer patients. ERJ Open Res *4,* 58. DOI: 10.1183/23120541.00058-2017

[144]: Cho BC, Kim D, Bearz A, Laurie SA, McKeage M, Borra G, Park K, Kim S, Ghosn M, Ardizzoni A, Maiello E, Greystoke A, Yu R, Osborne K, Gu W, Scott JW, Passos VQ, Lau YY, Wrona A (2017) ASCEND-8: A Randomized Phase 1 Study of Ceritinib, 450 mg or 600 mg, Taken with a Low-Fat Meal versus 750 mg in Fasted State in Patients with Anaplastic Lymphoma Kinase (ALK)-Rearranged Metastatic Non-Small Cell Lung Cancer (NSCLC). J Thorac Oncol *12,* 1357-1367. DOI: 10.1016/j.jtho.2017.07.005

[145]: Berdelou A, Borget I, Godbert Y, Nguyen T, Garcia M, Chougnet CN, Ferru A, Buffet C, Chabre O, Huillard O, Leboulleux S, Schlumberger M (2018) Lenvatinib for the Treatment of Radioiodine-Refractory Thyroid Cancer in Real-Life Practice. Thyroid *28,* 72-78. DOI: 10.1089/thy.2017.0205

[146]: Platini F, Cavalieri S, Alfieri S, Bergamini C, Resteghini C, Bottiglieri A, Colombo E, Mazzeo L, Licitra L, Paolini B, Seregni E, Locati LD (2021) Late toxicities burden in patients with radioiodine-refractory differentiated thyroid cancer treated with lenvatinib. Endocrine *73,* 641-647. DOI: 10.1007/s12020-021-02702-4

[147]: Motzer R, Alekseev B, Rha S, Porta C, Eto M, Powles T, Grünwald V, Hutson TE, Kopyltsov E, Méndez-Vidal MJ, Kozlov V, Alyasova A, Hong S, Kapoor A, Alonso Gordoa T, Merchan JR, Winquist E, Maroto P, Goh JC, Kim M, Gurney H, Patel V, Peer A, Procopio G, Takagi T, et al (2021) Lenvatinib plus Pembrolizumab or Everolimus for Advanced Renal Cell Carcinoma. N Engl J Med *384,* 1289-1300. DOI: 10.1056/NEJMoa2035716

[148]: Hutson TE, Michaelson MD, Kuzel TM, Agarwal N, Molina AM, Hsieh JJ, Vaishampayan UN, Xie R, Bapat U, Ye W, Jain RK, Fishman MN (2021) A Single-arm, Multicenter, Phase 2 Study of Lenvatinib Plus Everolimus in Patients with Advanced Non-Clear Cell Renal Cell Carcinoma. Eur Urol *80,* 162-170. DOI: 10.1016/j.eururo.2021.03.015

[149]: Goh MJ, Oh JH, Park Y, Kim J, Kang W, Sinn DH, Gwak G, Paik Y, Choi MS, Lee JH, Koh KC, Paik SW (2021) Efficacy and Safety of Lenvatinib Therapy for Unresectable Hepatocellular Carcinoma in a Real-World Practice in Korea. Liver Cancer *10,* 52-62. DOI: 10.1159/000512239

[150]: Rodríguez-Cid JR, Campos-Gomez S, García-Montes V, Magallanes-Maciel M, Flores-Mariñelarena RR, Fernández-Garibay VM, González-Espinoza IR, Ceja-García JP, Cázarez-Price JC, Martínez-Barrera L, Barriguete-Parra L, Zuloaga-Fernandez CJ, Kuri-Exsome R, Suárez-García D, Gonzalez-Villanueva JI, Flores-Anaya N, Acevedo-Delgado JA, Astorga-Ramos AM, Gerson-Cwilich R, Villalobos-Prieto A, Rodríguez-Silva C, Noriega-Iriondo MF, Vázquez-Cortés L, Perales-Rodríguez E, Acosta-Espinoza A, et al (2020) Real-World Evidence: Multicenter Efficacy and Toxicity Analysis of Nintedanib With Docetaxel as Second-Line Treatment in Mexican Patients With Advanced Lung Adenocarcinoma. JCO Glob Oncol *6,* 462-470. DOI: 10.1200/JGO.19.00330

[151]: Gottfried M, Bennouna J, Bondarenko I, Douillard J, Heigener DF, Krzakowski M, Mellemgaard A, Novello S, Orlov S, Summers Y, von Pawel J, Stöhr J, Kaiser R, Reck M (2017) Efficacy and Safety of Nintedanib Plus Docetaxel in Patients with Advanced Lung Adenocarcinoma: Complementary and Exploratory Analyses of the Phase III LUME-Lung 1 Study. Target Oncol *12,* 475-485. DOI: 10.1007/s11523-017-0517-2

[152]: Planchard D, Besse B, Groen HJM, Hashemi SMS, Mazieres J, Kim TM, Quoix E, Souquet P, Barlesi F, Baik C, Villaruz LC, Kelly RJ, Zhang S, Tan M, Gasal E, Santarpia L, Johnson BE (2022) Phase 2 Study of Dabrafenib Plus Trametinib in Patients With BRAF V600E-Mutant Metastatic NSCLC: Updated 5-Year Survival Rates and Genomic Analysis. J Thorac Oncol *17,* 103-115. DOI: 10.1016/j.jtho.2021.08.011

[153]: Ramalingam SS, Vansteenkiste J, Planchard D, Cho BC, Gray JE, Ohe Y, Zhou C, Reungwetwattana T, Cheng Y, Chewaskulyong B, Shah R, Cobo M, Lee KH, Cheema P, Tiseo M, John T, Lin M, Imamura F, Kurata T, Todd A, Hodge R, Saggese M, Rukazenkov Y, Soria J (2020) Overall Survival with Osimertinib in Untreated, EGFR-Mutated Advanced NSCLC. N Engl J Med *382,* 41-50. DOI: 10.1056/NEJMoa1913662

[154]: Finn RS, Rugo HS, Gelmon KA, Cristofanilli M, Colleoni M, Loi S, Schnell P, Lu DR, Theall KP, Mori A, Gauthier E, Bananis E, Turner NC, Diéras V (2021) Long-Term Pooled Safety Analysis of Palbociclib in Combination with Endocrine Therapy for Hormone Receptor-Positive/Human Epidermal Growth Factor Receptor 2-Negative Advanced Breast Cancer: Updated Analysis with up to 5 Years of Follow-Up. Oncologist *26,* e749-e755. DOI: 10.1002/onco.13684

[155]: Iwama E, Goto Y, Murakami H, Tsumura S, Sakashita H, Mori Y, Nakagaki N, Fujita Y, Seike M, Bessho A, Ono M, Nishitsuji M, Akamatsu H, Morinaga R, Akagi T, Shimose T, Tokunaga S, Yamamoto N, Nakanishi Y, Sugio K, Okamoto I (2020) Survival Analysis for Patients with ALK Rearrangement-Positive Non-Small Cell Lung Cancer and a Poor Performance Status Treated with Alectinib: Updated Results of Lung Oncology Group in Kyushu 1401. Oncologist *25,* 306-e618. DOI: 10.1634/theoncologist.2019-0728

[156]: Ou SI, Gadgeel SM, Barlesi F, Yang JC, De Petris L, Kim D, Govindan R, Dingemans A, Crino L, Léna H, Popat S, Ahn JS, Dansin E, Mitry E, Müller B, Bordogna W, Balas B, Morcos PN, Shaw AT (2020) Pooled overall survival and safety data from the pivotal phase II studies (NP28673 and NP28761) of alectinib in ALK-positive non-small-cell lung cancer. Lung Cancer *139,* 22-27. DOI: 10.1016/j.lungcan.2019.10.015

[157]: Mok T, Camidge DR, Gadgeel SM, Rosell R, Dziadziuszko R, Kim D, Pérol M, Ou S, Ahn JS, Shaw AT, Bordogna W, Smoljanović V, Hilton M, Ruf T, Noé J, Peters S (2020) Updated overall survival and final progression-free survival data for patients with treatment-naive advanced ALK-positive non-small-cell lung cancer in the ALEX study. Ann Oncol *31,* 1056-1064. DOI: 10.1016/j.annonc.2020.04.478

[158]: Berger T, Rozovski U, Moshe Y, Yaari S, Frisch A, Hellmann I, Apel A, Aviram A, Koren-Michowitz M, Yeshurun M, Ram R, Raanani P, Ofran Y, Wolach O (2019) Midostaurin in combination with intensive chemotherapy is safe and associated with improved remission rates and higher transplantation rates in first remission-a multi-center historical control study. Ann Hematol *98,* 2711-2717. DOI: 10.1007/s00277-019-03795-8

[159]: Szudy-Szczyrek A, Bachanek-Mitura O, Gromek T, Chromik K, Mital A, Szczyrek M, Krupski W, Szumiło J, Kanduła Z, Helbig G, Hus M (2021) Real-World Efficacy of Midostaurin in Aggressive Systemic Mastocytosis. J Clin Med *10,* 1109. DOI: 10.3390/jcm10051109

[160]: Hartmann K, Gotlib J, Akin C, Hermine O, Awan FT, Hexner E, Mauro MJ, Menssen HD, Redhu S, Knoll S, Sotlar K, George TI, Horny H, Valent P, Reiter A, Kluin-Nelemans HC (2020) Midostaurin improves quality of life and mediator-related symptoms in advanced systemic mastocytosis. J Allergy Clin Immunol *146,* 356-366.e4. DOI: 10.1016/j.jaci.2020.03.044

[161]: Hortobagyi GN, Stemmer SM, Burris HA, Yap Y, Sonke GS, Hart L, Campone M, Petrakova K, Winer EP, Janni W, Conte P, Cameron DA, André F, Arteaga CL, Zarate JP, Chakravartty A, Taran T, Le Gac F, Serra P, O'Shaughnessy J (2022) Overall Survival with Ribociclib plus Letrozole in Advanced Breast Cancer. N Engl J Med *386,* 942-950. DOI: 10.1056/NEJMoa2114663

[162]: Im S, Lu Y, Bardia A, Harbeck N, Colleoni M, Franke F, Chow L, Sohn J, Lee K, Campos-Gomez S, Villanueva-Vazquez R, Jung K, Chakravartty A, Hughes G, Gounaris I, Rodriguez-Lorenc K, Taran T, Hurvitz S, Tripathy D (2019) Overall Survival with Ribociclib plus Endocrine Therapy in Breast Cancer. N Engl J Med *381,* 307-316. DOI: 10.1056/NEJMoa1903765

[163]: Slamon DJ, Neven P, Chia S, Jerusalem G, De Laurentiis M, Im S, Petrakova K, Valeria Bianchi G, Martín M, Nusch A, Sonke GS, De la Cruz-Merino L, Beck JT, Ji Y, Wang C, Deore U, Chakravartty A, Zarate JP, Taran T, Fasching PA (2021) Ribociclib plus fulvestrant for postmenopausal women with hormone receptor-positive, human epidermal growth factor receptor 2-negative advanced breast cancer in the phase III randomized MONALEESA-3 trial: updated overall survival. Ann Oncol *32,* 1015-1024. DOI: 10.1016/j.annonc.2021.05.353

[164]: Molina AM, Hutson TE, Nosov D, Tomczak P, Lipatov O, Sternberg CN, Motzer R, Eisen T (2018) Efficacy of tivozanib treatment after sorafenib in patients with advanced renal cell carcinoma: crossover of a phase 3 study. Eur J Cancer *94,* 87-94. DOI: 10.1016/j.ejca.2018.02.009

[165]: Rini BI, Pal SK, Escudier BJ, Atkins MB, Hutson TE, Porta C, Verzoni E, Needle MN, McDermott DF (2020) Tivozanib versus sorafenib in patients with advanced renal cell carcinoma (TIVO-3): a phase 3, multicentre, randomised, controlled, open-label study. Lancet Oncol *21,* 95-104. DOI: 10.1016/S1470-2045(19)30735-1

[166]: Johnston S, O'Shaughnessy J, Martin M, Huober J, Toi M, Sohn J, André VAM, Martin HR, Hardebeck MC, Goetz MP (2021) Abemaciclib as initial therapy for advanced breast cancer: MONARCH 3 updated results in prognostic subgroups. NPJ Breast Cancer *7,* 80. DOI: 10.1038/s41523-021-00289-7

[167]: Goetz MP, Martin M, Tokunaga E, Park IH, Huober J, Toi M, Stoffregen C, Shekarriz S, Andre V, Gainford MC, Price GL, Johnston S (2020) Health-Related Quality of Life in MONARCH 3: Abemaciclib plus an Aromatase Inhibitor as Initial Therapy in HR+, HER2- Advanced Breast Cancer. Oncologist *25,* e1346-e1354. DOI: 10.1634/theoncologist.2020-0084

[168]: Rugo HS, Huober J, García-Sáenz JA, Masuda N, Sohn JH, Andre VAM, Barriga S, Cox J, Goetz M (2021) Management of Abemaciclib-Associated Adverse Events in Patients with Hormone Receptor-Positive, Human Epidermal Growth Factor Receptor 2-Negative Advanced Breast Cancer: Safety Analysis of MONARCH 2 and MONARCH 3. Oncologist *26,* e53-e65. DOI: 10.1002/onco.13531

[169]: Ascierto PA, Dummer R, Gogas HJ, Flaherty KT, Arance A, Mandala M, Liszkay G, Garbe C, Schadendorf D, Krajsova I, Gutzmer R, de Groot JWB, Loquai C, Gollerkeri A, Pickard MD, Robert C (2020) Update on tolerability and overall survival in COLUMBUS: landmark analysis of a randomised phase 3 trial of encorafenib plus binimetinib vs vemurafenib or encorafenib in patients with BRAF V600-mutant melanoma. Eur J Cancer *126,* 33-44. DOI: 10.1016/j.ejca.2019.11.016

[170]: Tabernero J, Grothey A, Van Cutsem E, Yaeger R, Wasan H, Yoshino T, Desai J, Ciardiello F, Loupakis F, Hong YS, Steeghs N, Guren TK, Arkenau H, Garcia-Alfonso P, Elez E, Gollerkeri A, Maharry K, Christy-Bittel J, Kopetz S (2021) Encorafenib Plus Cetuximab as a New Standard of Care for Previously Treated BRAF V600E-Mutant Metastatic Colorectal Cancer: Updated Survival Results and Subgroup Analyses from the BEACON Study. J Clin Oncol *39,* 273-284. DOI: 10.1200/JCO.20.02088

[171]: Huber RM, Hansen KH, Paz-Ares Rodríguez L, West HL, Reckamp KL, Leighl NB, Tiseo M, Smit EF, Kim D, Gettinger SN, Hochmair MJ, Kim S, Langer CJ, Ahn M, Kim ES, Kerstein D, Groen HJM, Camidge DR (2020) Brigatinib in Crizotinib-Refractory ALK+ NSCLC: 2-Year Follow-up on Systemic and Intracranial Outcomes in the Phase 2 ALTA Trial. J Thorac Oncol *15,* 404-415. DOI: 10.1016/j.jtho.2019.11.004

[172]: Camidge DR, Kim HR, Ahn M, Yang JCH, Han J, Hochmair MJ, Lee KH, Delmonte A, Garcia Campelo MR, Kim D, Griesinger F, Felip E, Califano R, Spira AI, Gettinger SN, Tiseo M, Lin HM, Liu Y, Vranceanu F, Niu H, Zhang P, Popat S (2021) Brigatinib Versus Crizotinib in ALK Inhibitor-Naive Advanced ALK-Positive NSCLC: Final Results of Phase 3 ALTA-1L Trial. J Thorac Oncol *16,* 2091-2108. DOI: 10.1016/j.jtho.2021.07.035

[173]: Mok TS, Cheng Y, Zhou X, Lee KH, Nakagawa K, Niho S, Chawla A, Rosell R, Corral J, Migliorino MR, Pluzanski A, Noonan K, Tang Y, Pastel M, Wilner KD, Wu Y (2021) Updated Overall Survival in a Randomized Study Comparing Dacomitinib with Gefitinib as First-Line Treatment in Patients with Advanced Non-Small-Cell Lung Cancer and EGFR-Activating Mutations. Drugs *81,* 257-266. DOI: 10.1007/s40265-020-01441-6

[174]: Perl AE, Larson RA, Podoltsev NA, Strickland S, Wang ES, Atallah E, Schiller GJ, Martinelli G, Neubauer A, Sierra J, Montesinos P, Récher C, Yoon S, Hosono N, Onozawa M, Chiba S, Kim H, Hasabou N, Lu Q, Tiu R, Levis MJ (2022) Follow-up of patients with R/R FLT3-mutation-positive AML treated with gilteritinib in the phase 3 ADMIRAL trial. Blood *139,* 3366-3375. DOI: 10.1182/blood.2021011583

[175]: Hong DS, DuBois SG, Kummar S, Farago AF, Albert CM, Rohrberg KS, van Tilburg CM, Nagasubramanian R, Berlin JD, Federman N, Mascarenhas L, Geoerger B, Dowlati A, Pappo AS, Bielack S, Doz F, McDermott R, Patel JD, Schilder RJ, Tahara M, Pfister SM, Witt O, Ladanyi M, Rudzinski ER, Nanda S, et al (2020) Larotrectinib in patients with TRK fusion-positive solid tumours: a pooled analysis of three phase 1/2 clinical trials. Lancet Oncol *21,* 531-540. DOI: 10.1016/S1470-2045(19)30856-3

[176]: Peters S, Shaw AT, Besse B, Felip E, Solomon BJ, Soo RA, Bearz A, Gadgeel SM, Lin C, Kao S, Seto T, Masters ET, Abbattista A, Clancy JS, Thurm H, Reisman A, Peltz G, Ross Camidge D (2020) Impact of lorlatinib on patient-reported outcomes in patients with advanced ALK-positive or ROS1-positive non-small cell lung cancer. Lung Cancer *144,* 10-19. DOI: 10.1016/j.lungcan.2020.02.011

[177]: Felip E, Shaw AT, Bearz A, Camidge DR, Solomon BJ, Bauman JR, Bauer TM, Peters S, Toffalorio F, Abbattista A, Thurm H, Peltz G, Wiltshire R, Besse B (2021) Intracranial and extracranial efficacy of lorlatinib in patients with ALK-positive non-small-cell lung cancer previously treated with second-generation ALK TKIs. Ann Oncol *32,* 620-630. DOI: 10.1016/j.annonc.2021.02.012

[178]: Martin M, Holmes FA, Ejlertsen B, Delaloge S, Moy B, Iwata H, von Minckwitz G, Chia SKL, Mansi J, Barrios CH, Gnant M, Tomašević Z, Denduluri N, Šeparović R, Gokmen E, Bashford A, Ruiz Borrego M, Kim S, Jakobsen EH, Ciceniene A, Inoue K, Overkamp F, Heijns JB, Armstrong AC, Link JS, et al (2017) Neratinib after trastuzumab-based adjuvant therapy in HER2-positive breast cancer (ExteNET): 5-year analysis of a randomised, double-blind, placebo-controlled, phase 3 trial. Lancet Oncol *18,* 1688-1700. DOI: 10.1016/S1470-2045(17)30717-9

[179]: Chan A, Moy B, Mansi J, Ejlertsen B, Holmes FA, Chia S, Iwata H, Gnant M, Loibl S, Barrios CH, Somali I, Smichkoska S, Martinez N, Alonso MG, Link JS, Mayer IA, Cold S, Murillo SM, Senecal F, Inoue K, Ruiz-Borrego M, Hui R, Denduluri N, Patt D, Rugo HS, et al (2021) Final Efficacy Results of Neratinib in HER2-positive Hormone Receptor-positive Early-stage Breast Cancer From the Phase III ExteNET Trial. Clin Breast Cancer *21,* 80-91.e7. DOI: 10.1016/j.clbc.2020.09.014
